# Bacterial Gut Microbiota and Infections During Early Childhood

**DOI:** 10.3389/fmicb.2021.793050

**Published:** 2022-01-05

**Authors:** Sergio George, Ximena Aguilera, Pablo Gallardo, Mauricio Farfán, Yalda Lucero, Juan Pablo Torres, Roberto Vidal, Miguel O’Ryan

**Affiliations:** ^1^Host-Pathogen Interaction Laboratory, Microbiology and Mycology Program, ICBM, Faculty of Medicine, University of Chile, Santiago, Chile; ^2^School of Medicine, Faculty of Medicine, University of Chile, Santiago, Chile; ^3^Department of Pediatrics and Pediatric Surgery, Dr. Luis Calvo Mackenna Hospital, Faculty of Medicine, University of Chile, Santiago, Chile; ^4^Department of Pediatrics and Pediatric Surgery, Dr. Roberto del Río Hospital, Faculty of Medicine, University of Chile, Santiago, Chile; ^5^Microbiology and Mycology Program, ICBM, Faculty of Medicine, University of Chile, Santiago, Chile; ^6^Millennium Institute on Immunology and Immunotherapy, Faculty of Medicine, University of Chile, Santiago, Chile; ^7^ANID - Millennium Science Initiative Program – Millennium Nucleus in the Biology of Intestinal Microbiota, Santiago, Chile

**Keywords:** gut microbiota, necrotizing enterocolitis, *Clostridioides difficile*, rotavirus, norovirus, diarrheagenic *Escherichia coli* (DEC), RSV, childhood infections

## Abstract

Gut microbiota composition during the first years of life is variable, dynamic and influenced by both prenatal and postnatal factors, such as maternal antibiotics administered during labor, delivery mode, maternal diet, breastfeeding, and/or antibiotic consumption during infancy. Furthermore, the microbiota displays bidirectional interactions with infectious agents, either through direct microbiota-microorganism interactions or indirectly through various stimuli of the host immune system. Here we review these interactions during childhood until 5 years of life, focusing on bacterial microbiota, the most common gastrointestinal and respiratory infections and two well characterized gastrointestinal diseases related to dysbiosis (necrotizing enterocolitis and *Clostridioides difficile* infection). To date, most peer-reviewed studies on the bacterial microbiota in childhood have been cross-sectional and have reported patterns of gut dysbiosis during infections as compared to healthy controls; prospective studies suggest that most children progressively return to a “healthy microbiota status” following infection. Animal models and/or studies focusing on specific preventive and therapeutic interventions, such as probiotic administration and fecal transplantation, support the role of the bacterial gut microbiota in modulating both enteric and respiratory infections. A more in depth understanding of the mechanisms involved in the establishment and maintenance of the early bacterial microbiota, focusing on specific components of the microbiota-immunity-infectious agent axis is necessary in order to better define potential preventive or therapeutic tools against significant infections in children.

## Introduction

Development in molecular microbiology techniques, sequencing platforms and bioinformatics during the past decade have allowed us to expand our knowledge on microbiota composition, dynamics, and impact on human health and disease. We now know that the most abundant and diverse human microbiota niche is harbored in the gastrointestinal tract. Moreover, during the first 5 years of childhood, the gut microbiota (GM) undergoes major changes as it progresses toward an “adult-like, more stable community.” This GM plays a role in the development of the enteric immune and nervous systems, nutrient metabolism, and interactions with infectious agents, among other functions. The GM can affect the occurrence and course of systemic diseases, not only during childhood, but also later in life, including autism spectrum disorders (Li et al., 2019), attention deficit hyperactivity disorder (Boonchooduang et al., 2020), asthma and allergies (Kim et al., 2019), obesity (Muscogiuri et al., 2019), and autoimmune disorders (Russell et al., 2019), among others. For better comprehension we include Table 1 with basic terms needed for a proper understanding of the topics discussed in this review.

**TABLE 1:** Definition of relevant terms included in microbiota studies, and acronyms used in this review.

Term	Definition	References
Relative abundance	Quantitative measure of the number of organisms, operational taxonomic units (OTUs), operational phylogenetic units (OPUs), amplicon sequence variants (ASVs) or sequences detected in a sample in relation to all others in that sample (e.g., If there are 100 organisms in a sample, and 20 are identified as *E. coli*, the relative abundance of *E. coli* is 20%)	Tyler et al., 2014; Yarza et al., 2014; Callahan et al., 2017
Richness	Number of unique organisms detected in a specific sample.	Tyler et al., 2014; McBurney et al., 2019
Diversity	Estimate incorporating species richness and abundance to measure the microbial variability either within (α) or between (β) samples.	Tyler et al., 2014
Enterotype	Classification of organisms into **c**ategories or clusters based on a process of stratification of microbiota to reduce global variation into a few categories driven by discriminative genera.	Cheng and Ning, 2019
Healthy microbiota and dysbiosis	A healthy microbiota can be described in terms of ecological stability (i.e., the ability to resist community structure change under stress or to rapidly return to baseline following a stress-related change) and by an idealized (presumably health-associated) composition or a desirable functional profile. Dysbiosis can be defined as an alteration in the microbiome from the normal or healthy state. For the purpose of this review, as microbiota composition is dependent on multiple variables, a healthy microbiota is considered to be the group cataloged as “healthy controls” in each study discussed.	Backhed et al., 2012; Tyler et al., 2014; McBurney et al., 2019

**Acronym**	**Meaning**	

ALRI	Acute lower respiratory infection	
AMP	Antimicrobial peptides	
AMR	Antimicrobial resistance	
ARI	Acute respiratory infection	
AURI	Acute upper respiratory infection	
BGM	Bacterial gut microbiota	
CD	*Clostridiodes difficile*	
DEC	Diarrheagenic *Escherichia coli*	
EAEC	Enteroaggregative *E. coli*	
EHEC	Enterohemorrhagic *Escherichia coli*	
EIEC	Enteroinvasive *E. coli*	
EPEC	Enteropathogenic *E. coli*	
ETEC	Enterotoxigenic *E. coli*	
GF mice	Germ free mice	
GM	Gut microbiota	
IFN	Interferon	
LPS	Lipopolysaccharide	
MAMP	Microbiota- associated molecular patterns	
NEC	Necrotizing enterocolitis	
NV	Norovirus	
OPU	Operational phylogenetic unit	
OTU	Operational taxonomic unit (OTUs)	
PAMP	Pathogens-associated molecular patterns	
PRR	Pattern recognition receptor	
RSV	Respiratory syncytial virus	
RV	Rotavirus	
SCFA	Short chain fatty acids	
SFB	Segmented filamentous bacteria	
STEC	Shiga toxin-producer *Escherichia coli*	
TLR/NLR	Toll-like receptor/NOD-like receptor	

Though bacteria dominate gut microbial communities, GM is also composed of viruses, fungus, parasites and archaea, denominated Virome, Mycobiome, Parasitome, and Archaeome, respectively (Vemuri et al., 2020). Methodological difficulties associated with their study (including lack of updated bioinformatic tools and reference databases) have most likely downplayed our understanding of their true role in GM. Recent advances in next generation sequencing technologies have reflected on increasing literature, especially on gut virome development from childhood to adulthood and its role in gut homeostasis and immune-related diseases including childhood infectious diseases, as extensively reviewed (Tiew et al., 2020; Vemuri et al., 2020; Fulci et al., 2021; Townsend et al., 2021; Wu et al., 2021). Briefly, adult gut virome is mainly composed by bacteriophages, and in lower proportion by DNA and RNA eukaryotic viruses. Development of virome during childhood is dynamic and occurs in a stepwise manner, with initial colonization of bacteriophages during the first weeks of life (after pioneer bacteria colonization in the newborn gut) (Liang et al., 2020), a progressive increase of eukaryotic viruses during first 2 years of life (Lim et al., 2015) together with an expansion of bacterial abundance and richness, and a later predominance of bacteriophages toward an adult-like composition (Gregory et al., 2020). Gut mycobiome (Strati et al., 2016), and archaeome (van de Pol et al., 2017; Pausan et al., 2019) composition in children have been explored, but their trajectories during the first years of life are not deeply understood at the moment. For parasites, some studies have evaluated the presence of commensal parasites in children and their effects on bacterial GM (BGM) composition (Alzate et al., 2020), but an exhaustive “omic” approach to understand the parasitome relevance during childhood is still needed.

This review focuses on the dynamics of BGM during childhood, and their relationships with the most common gastrointestinal and respiratory infections, as well as with two well identified dysbiosis-related diseases: necrotizing enterocolitis (NEC) and *Clostridioides difficile* infection.

## Bacterial Gut Microbiota Dynamics and Modulation During the First 5 Years of Life

Human gut bacterial communities in healthy adults are highly diverse, with each individual harboring over 100 trillion bacteria, belonging to over 150 different species. The BGM during adulthood is dominated by the phyla Firmicutes (e.g., *Lactobacillus*, *Bacillus*, and *Clostridioides*) and Bacteroidetes (e.g., *Bacteroides*), with lower abundances of Proteobacteria (e.g., *Escherichia*) and Actinobacteria (e.g., *Bifidobacterium*) (Arumugam et al., 2011; Rinninella et al., 2019). The establishment of this “mature microbiota” begins during early childhood and is influenced by prenatal and postnatal factors (Roswall et al., 2015; Chu et al., 2019).

Several studies have questioned whether the in-uterus environment is sterile. The presence of bacteria in amniotic fluid originating from both the maternal genitourinary tract and oral cavity was initially demonstrated in preterm infants, suggesting that ascending (from the genitourinary tract) and hematogenous (from the oral cavity) bacterial dissemination causes intra-amniotic colonization/infection (Romero et al., 2014). There is additional conflicting evidence, as some studies report the detection of bacteria in the placenta (Aagaard et al., 2014), amniotic fluid (Rautava et al., 2012) and the umbilical cord (Jimenez et al., 2005) in women with healthy term pregnancies, as well as in the meconium of term newborns (Su et al., 2018), while other studies report a sterile in-uterus environment in this context (Lim et al., 2018; Rehbinder et al., 2018). Thus, the role of “*in utero*” colonization is currently uncertain, this may in part be due to the lack of evidence of bacterial viability beyond detection of bacterial DNA (Perez-Munoz et al., 2017).

Delivery mode is a major determinant of BGM composition during early infancy. Vaginal delivery is followed by newborn gut colonization with the mother’s vaginal microbiota, mainly composed of *Prevotella* and *Lactobacillus* spp. (Dominguez-Bello et al., 2010), while the microbiota of infants delivered through cesarean section resembles that of the mother’s skin microbiota, and of the nosocomial environment (Dominguez-Bello et al., 2010; Shao et al., 2019). These differences are most distinct during the first 6 months of life (Rutayisire et al., 2016).

Breastfeeding is another key factor in determining the microbiota composition during early childhood (Ho et al., 2018; Ossa et al., 2018). A recent study showed that receiving breast milk (either exclusive or partial) was the most significant factor associated with gut microbiota structure from months 3 to 14 of life, and was associated with the presence of *Bifidobacterium* species and the species *Lactobacillus rhamnosus* and *Staphylococcus epidermidis* (Stewart et al., 2018). Both *Bifidobacterium* and *Lactobacillus* spp. are present in human milk and *S. epidermidis* colonize maternal skin; and their metabolic functions in falta una palabra aqui are related to breastfeeding, as *Bifidobacterium* species metabolize human milk oligosaccharides (HMOs) (James et al., 2019) and *Lactobacillus* species including *L. rhamnosus* can metabolize lactose (Lubiech and Twaruzek, 2020). Cessation of breastfeeding seems to be the major determinant in the “maturation” of the gut microbiota, characterized by an increase in Firmicutes (Stewart et al., 2018), concordant with other studies showing an increase specifically in the *Lachnospiraceae* and *Ruminococcaceae* families (Laursen et al., 2016), which can metabolize plant-derived complex carbohydrates introduced with solid foods (Flint et al., 2012). After 6 months of age, the composition of complementary diet also determines the BGM evolution, e.g., the progression to a higher protein and fiber diet is associated with an increase in microbial α-diversity (Laursen et al., 2016).

Overall, a history of breastfeeding seems to be more influential than the mode of delivery in determining long-term BGM composition, e.g., the lack of breastfeeding during infancy is associated with higher degrees of the genus *Bacteroides* in stool during childhood and adolescence (Cioffi et al., 2020). However, the impact of these findings on a child’s clinical outcomes is not entirely elucidated.

Maternal diet may also influence the composition of infant gut-microbiota during the first 6 weeks of life, especially in the case of a high-fat maternal diet (Chu et al., 2016), fruit intake and dairy intake (Lundgren et al., 2018; Maher et al., 2020). Maternal diet is also associated with changes in mother’s milk microbiota, which may influence the BGM in the child (Babakobi et al., 2020). Overall, these changes in BGM composition reflect a gradual functional specialization in parallel to the nutritional changes over time, in order to deal with the substrates provided by changing diets, as extensively reviewed by other authors (Derrien et al., 2019).

Geographic differences in BGM composition during the first years of life have been described, most likely due in part to differences in diet and socioeconomic status; e.g., 6-month-old children from Malawi have increased abundance of *Bifidobacterium* group, *Bacteroides-Prevotella* group, and *Clostridium histolyticum* group compared to children from Finland (Grzeskowiak et al., 2012); while 1–6 years old children from Burkina Faso have enrichment in Bacteroidetes and depletion in Firmicutes compared to European children (De Filippo et al., 2010). African children have a diet based primarily on fiber and complex carbohydrates, which results in higher levels of fecal short-chain fatty acids (SCFA), likely reflecting the ability of their microbiota to degrade these complex carbohydrates. Conversely, European children have a characteristically “western diet” (high in animal protein, sugar, starch, fat, and low in fiber), associated with low fecal SCFA levels; thus they develop a BGM more suited to metabolize simple carbohydrates, animal fat and protein (De Filippo et al., 2017). These differences suggest a tight relationship among BGM evolution, diet and functionality.

Exposure to antibiotics can modulate BGM composition from gestation onwards. The use of intrapartum antibiotics for prophylaxis against group B *Streptococcus* infection was associated with lower bacterial diversity, lower abundance of Actinobacteria and Bacteroidetes phyla; and higher abundance of Proteobacteria phylum and *Enterobacteriaceae* or *Streptococcaceae* families in the BGM of newborns, compared to those delivered by mothers not receiving antibiotic prophylaxis (Aloisio et al., 2016). These changes seem to be more prominent, lasting up to day 30 of life in breastfed infants (Mazzola et al., 2016). When the delivery mode is concomitantly analyzed, intrapartum antibiotic use is associated with a lower abundance of the genus *Bacteroides* and *Parabacteroides* and higher abundance of *Enterococcus* and *Clostridium* levels at 3 months of life irrespective of delivery mode; changes are more prominent and persist up to 12 months of life in children born by emergency cesarean section, especially in those who did not receive exclusive breastfeeding during first 3 months of life (Azad et al., 2016).

The use of systemic antibiotics during the neonatal period has been associated with disruption of normal microbiota colonization in both preterm and term newborns, with lower rates of commensal bacteria such as the genus *Bifidobacterium* (Tanaka et al., 2009) and phylum Bacteroidetes (Eck et al., 2020) and increased rates of potentially pathogenic bacteria such as the genus *Enterobacter* from the first weeks of life (Greenwood et al., 2014). Changes in BGM diversity and composition related to the early use of narrow-spectrum antibiotics persists up to 6 months of life (Tapiainen et al., 2019). In addition, perinatal antibiotic use is associated with increased antimicrobial resistance (AMR) genes in stools of both preterm and term newborns (Gibson et al., 2016; Tapiainen et al., 2019).

Throughout the first 3 years of life, children with repeated short courses of oral antibiotics (prescribed mainly for “respiratory infections”) have decreased BGM in terms of both bacterial species and strains, and increased variability in composition compared to children not receiving antibiotics (Yassour et al., 2016). Expectedly, detection of AMR genes increases after antibiotic intake; chromosomal genes increase rapidly and decrease after antibiotics are ceased, while some episomally codified AMR genes do not decrease until several months after antibiotic consumption (Yassour et al., 2016). Moreover, macrolides but not penicillin consumption has been associated with a distinct microbiota composition at the phylum level, characterized by an increased abundance of the phyla Bacteroidota and Proteobacteria, and decreased abundance of Actinobacteria (Korpela et al., 2016) in children 2–7 years old. In addition, macrolide use was associated with a long-term (12–24 months) reduction in microbial richness, but a transitory effect on macrolide resistance genes and culture-based macrolide resistance in the gut which increased immediately after macrolide intake and returned to low levels at 6–12 months (Korpela et al., 2016).

Interestingly, mass azithromycin distribution in biannual courses in Nigerian preschool children (1–59 months) reduced all-causes of mortality by 13.5% compared to placebo (Keenan et al., 2018), and the effect on mortality was higher in children 1–5 months of age (24.9% lower mortality). Microbiota analysis of these children showed that the relative abundances of two *Campylobacter* species (which cause diarrhea and are susceptible to azithromycin) along with another 33 gut bacteria were significantly reduced at 24-months of follow-up. This suggests a potential short-term benefit of macrolides in the control of enteric infectious diseases; however, macrolide resistance genes increased during the following 6 months (Doan et al., 2019).

In summary, normal BGM colonization begins *in utero* or during birth, and evolves during the first years of life influenced by several environmental and host factors which finally determine a BGM’s individual pattern. As recently described in a large Swedish cohort, BGM progression during this period is characterized by increasing α-diversity as children get older, with major shifts in composition between 4 and 12 months, resembling a more adult microbiota as the children reach 3–5 years of age (Roswall et al., 2021). Based on this cohort of children, four characteristic trajectories were defined based on relative abundance of single genera at different time points: (1) a first peak at 4 months of *Bifidobacterium, Enterococcus, Streptococcus, Lactobacillus* and Enterobacteriaceae; (2) a second peak at 12 months, mainly in *Ruminococcus* abundance; (3) a rapid increase in *Bacteroides* between 4 and 12 months of age and relative stability after 3 years of age; and (4) an increase in *Methanobrevibacter*, *Desulfovibrio*, *Bilophila*, and some *Clostridia* after 12 months followed by additional increases after 3 years. Noticeably, despite the fact that these major and critical changes are observed during these first years of life, at 5 years of age the childhood BGM composition was still significantly different compared with their mothers and other unrelated adults (Roswall et al., 2021), suggesting that the BGM continues to evolve during childhood and future life. Whether these results are also observed in other geographical regions, remains to be seen.

## Mechanisms Involved in Bacterial Gut Microbiota–Pathogen Interactions

The eubiotic BGM plays an important role in sustaining a “state of good health” as well as in modulating pathogenesis of several diseases, including susceptibility to infections and clinical outcomes after infection has occurred (Harris et al., 2017b; Gaufin et al., 2018; Libertucci and Young, 2019).

The pathophysiology behind BGM-infectious pathogen interactions is influenced by host factors. Two fundamental pathways are involved: one direct, mediated by microbiota interactions with non-commensal agents, and another indirect, via microbiota-mediated immune system modulation (Libertucci and Young, 2019).

### Microbiota–Pathogen Interactions

Commensal bacteria limit (or enhance) pathogen colonization through direct bactericidal/bacteriostatic or stimulatory effects (by direct binding or mediated by metabolites), as well as through competition for nutrients and specific resources required for infection, in addition to modification of gut mucosa sugars and/or receptors. Key factors involved in microbiota–pathogen interactions are summarized in Table 2.

**TABLE 2:** Mechanisms involving direct interaction between the gut microbiota and pathogens.

Mechanism	Description	References
Availability of host sugars	− Pathogens, such as *Salmonella typhimurium* and *Clostridioides difficile*, catabolize free sialic acid and fucose in the colonic lumen as a source of energy. Commensal *Bacteroides thetaiotaomicron* codifies sialidases, promoting free sialic acid production and the release of fucose from mucus. These microbiota-liberated host sugars facilitate post-antibiotic expansion of *S. typhimurium* and *C. difficile* in mice models.	Pacheco et al., 2012; Ng et al., 2013; Baumler and Sperandio, 2016
	− Enterohemorrhagic *Escherichia coli* (EHEC) can sense fucose released by the effect of *B. thetaiotaomicron*. Fucose-sensing mechanisms allow EHEC to express virulence factors and colonize the intestine.	
Gut microbiota- mediated glycan modification	− Soluble factors produced by *Lactobacillus casei* and *B. thetaiotaomicron* can alter cell surface glycoproteins, which results in decreased binding of rotavirus (RV) to intestinal cells.	Varyukhina et al., 2012
Direct binding of gut microbiota bacteria and viral pathogens	− Norovirus (NV) binds to HBGA-like carbohydrates expressed on the surface of the gut bacteria *E. coli* and *Enterobacter cloacae.* This binding allows NV infection of target cells (B cells), and also protects NV from heat stress in *in vitro* models.	Miura et al., 2013; Jones et al., 2014; Li et al., 2015; Shi et al., 2019
	− Rotavirus (RV) infectivity is reduced by segmented filamentous bacteria in *in vitro* studies.	
Direct effect of microbiota- derived metabolites on pathogens	− Exposure of *Salmonella typhimurium* to short chain fatty acid (SCFA) acetate concentrations found in the ileum enhances type III secretion system (T3SS) expression; while propionate and butyrate levels in the colon inhibit expression. In EHEC infections, colonic butyrate levels enhance T3SS expression.	Baumler and Sperandio, 2016; Li Z. et al., 2018
	− Bacteriocins produced by the microbiota act directly on pathogens by limiting infection (e.g., Nisin produced by *Lactococcus lactis* is a pore-forming bacteriocin for *Salmonella enterica*, *Staphylococcus aureus*, and *Bacillus cereus*).	
	− Other gut microbiota metabolites (such as microbial amino acids, vitamins, and quorum sensing autoinducers) act on pathogens by limiting or promoting infection.	

### Microbiota-Mediated Immune System Modulation

Experiments in germ-free (GF) mice have provided significant information on the role of the BGM in the development and functionality of the immune system, as summarized in Table 3. The microbiota and key components of the innate and adaptive immune system interact in a bidirectional manner, e.g., the BGM composition determines the development of either effective or defective innate and adaptive immune system components, and, specific components of the immune system contribute to either maintain or break BGM homeostasis. Development of the BGM is related to immune system maturation, and the first weeks of life seem to be critical as evaluated in animal models. Based on the age-dependant effect of the BGM restoration in GF animals on several immune components (iNKT, Treg, IgE, and TLR signaling on epithelial cells), Gensollen et al. (2016) postulated the term “window of opportunity” related to the period during early infancy where BGM can still be modified, otherwise resulting in permanent immune alterations. In humans, this model could explain the association of BGM alterations during early childhood with future immune-mediated chronic diseases including allergy, asthma, undernutrition, or obesity and inflammatory bowel disease (Arrieta et al., 2014). In the following sections, we will analyze the association of BGM composition during this apparently critical period with common infections during childhood.

**TABLE 3:** Recognized interactions between the immune system and the gut microbiota.

Immune system component	Function	Interactions with the microbiota	References
**Innate immune response**			
Mucus	Physical barrier	− Germ-free (GF) mice display alterations in the composition and structure of the mucus layer compared to normally-colonized mice.	Petersson et al., 2011; Johansson et al., 2015; Paone and Cani, 2020
		− Bacterial factors (LPS, peptidoglycan) promote mucus secretion and restoration in GF mice.	
		− Host glycosylation patterns influence the composition of mucus-associated bacteria.	
		− “Mucolytic bacteria” use mucins as nutrients.	
Tight junctions	Restrict paracellular permeability to pathogens	Gut microbiota perturbation induced by a high fat diet and antibiotic use is associated with reduced expression of tight junction proteins in mice, and increased intestinal permeability.	Cani et al., 2009; Ahmad et al., 2017; Feng Y. et al., 2019
Pattern recognition receptors (PRR)	Innate immune-system receptors recognizing pathogens or microbiota- associated molecular patterns (PAMPs-MAMPs) activating immune responses or maintaining gut homeostasis.	− There are bidirectional interactions between the microbiota PAMPs and PRRs.	Bouskra et al., 2008; Petnicki-Ocwieja et al., 2009; Chassin et al., 2010; Stockinger et al., 2011; Carvalho et al., 2012
		− Microbiota recognition by PRRs is essential in immune system development [e.g., antimicrobial peptide (AMP) production, epithelial proliferation and gut-associated lymphoid tissue development].	
		− PRR interactions with the microbiota maintain microbiota homeostasis (e.g., NOD1-defficient mice display an increase in *Clostridiales*, *Bacteroides* and *Enterobacteriaceae*; NOD2-deficient mice display an increase in ileal *Bacteroidota* and *Firmicutes*).	
		− Altered PRR detection in the gut microbiota is associated with increased intestinal inflammation in response to pathogens, and may lead to chronic inflammation-associated diseases, such as cancer and metabolic syndromes.	
		− In vaginally delivered newborn mice, downregulation of Toll-like receptors (TLR) signaling in intestinal epithelial cells is critical in stablishing gut tolerance to bacteria during this period, which allows the gut colonization.	
Antimicrobial peptides (AMP)	Limit pathogen interaction with the epithelia	− BGM (e.g., *Bacteroides thetaiotaomicron*) enhance AMP production by epithelial cells. Flagellin stimulate TLR-5 in dendritic cells and epithelial cells to enhance the epithelial expression of AMPs.	Cash et al., 2006; Kinnebrew et al., 2010; Levy et al., 2015; Sivieri et al., 2017; Zhao et al., 2018
		− SCFA (mainly butyrate, acetate, and propionate) are metabolites produced by certain gut-microbiota components from metabolism of dietary fiber. SCFAs produced by the microbiota induce intestinal epithelial cell production of AMPs.	
		− AMPs regulate the quantity and composition of intestinal microbiota.	

**Adaptive immune response**			

IgA- B cell response	Secretory immunoglobulin	− Enteric microbiota induce mucosal immune system maturation and production of high levels of secretory IgA.	Pabst et al., 2016
		− In pIgR-deficient mice (deficient in all secretory Igs): presence of secretory IgA affects microbial fitness and thereby microbiota composition.	
IgE - B cell response	Immunoglobulin E, related to Th2 and allergic response.	− Germ-free mice have an elevated systemic IgE response, driven by a B-cell isotype change in mucosal lymphoid tissues, in a LTCD4 and IL-4-dependant manner.	Cahenzli et al., 2013
		− Colonization of GF mice during the first 2 weeks of life (but not after) restores IgE levels permanently until adulthood. This is dependent on a high BGM diversity.	
Regulatory T cell (TReg) response	Maintains immune balance by limiting effector immune cell responses. In infections, they can have either a potentially protective role, by limiting pathogen-induced immunopathology, or detrimental by limiting effector cell-mediated eradication of pathogen.	− Microbiota can induce differentiation of naïve-T cells to peripheral TReg cells, mediated by SCFA production from dietary fiber metabolism (e.g., by Clostridia clusters XIVa, IV, and XVIII), or by bacterial polysaccharides (e.g., from *Bacteroides fragilis*).	Belkaid, 2007; Arpaia et al., 2013; Furusawa et al., 2013
Th17 cell response	Potentially protective mainly against bacterial and fungal, extracellular infections.	− Segmented filamentous bacteria (and their flagellin) from gut microbiota induce Th17 cell differentiation.	Ivanov et al., 2009; Honda and Littman, 2016; Wang et al., 2019
Invariant natural killer T cells (iNKT)	Subset of T lymphocytes, harboring T-cell receptors (TCR) recognizing glycosphingolipid presented by CD1d. iNKT activation can produce either a Th1 or Th2 cytokine response.	− GF mice have diminished and hyporesponsive iNKT in peripheral tissues, but augmented colonic iNKT associated with colitis.	Olszak et al., 2012; Wingender et al., 2012; An et al., 2014; Hapil and Wingender, 2018
		− Colonization with complete BGM, *Bacteroides fragilis* or its related sphingolipids during first 2 weeks (but not thereafter) reverts iNKT accumulation in colon.	
		− Several commensal bacteria have sphingolipids that activate iNKT, including *Lactobacillus casei, Prevotella copri, Bacteroides fragilis*, and *Bacteroides vulgatus.*	

## Gut Microbiota and Specific Gastrointestinal Infectious Diseases

### Necrotizing Enterocolitis

Necrotizing enterocolitis is characterized by intestinal inflammation and necrosis that can progress to systemic infection, multiorgan failure, death (Tanner et al., 2015), and long-term neurological complications (Rees et al., 2007). Prematurity is the main risk factor, and additional factors include lack of human milk feeding (Cacho et al., 2017), meconium aspiration syndrome, postnatal asphyxia, congenital heart disease (Lu et al., 2017), and aberrant microbial colonization and infections (Neu and Pammi, 2018). Microorganisms that potentially play a role include: Gram-negative enteric bacteria (*Escherichia coli*, *Klebsiella* spp., and *Pseudomonas aeruginosa*), Gram-positive bacteria (*Enterococcus, Staphylococcus aureus*, and *S. epidermidis*), Viruses (rotavirus, norovirus, astrovirus, cytomegalovirus, and echovirus), and fungus (*Candida* spp.) (Coggins et al., 2015). Studies based on several different animal models have shown that key factors in NEC pathogenesis are immune response and the coexistence of multifactorial dysbiosis and an altered gut barrier. This alteration is characterized by an increased TLR4 response to gut bacteria, decreased AMPs and mucin production, and impaired production of certain growth factors and cytokines (epidermal growth factor, TGF-β and IL-10, among others). The result is intestinal bacterial translocation, a local and systemic pro-inflammatory response, and gut mucosal damage and necrosis (Tanner et al., 2015).

In humans, prematurity itself is related to an altered BGM development pattern compared with term infants. This altered development pattern is also influenced by other prematurity-related factors, e.g., gastrointestinal and immune system immaturity, early antibiotics use, long-term hospitalization, mechanical ventilation and parenteral nutrition (Tirone et al., 2019). In comparison to term infants, the preterm BGM displays an evolutionary pattern characterized by an initial predominance of the class Bacilli, followed by predominance of the class Gammaproteobacteria, and later on by Clostridia (La Rosa et al., 2014); in a minor taxonomic level, a progression in four phases characterized by dominance of *Staphylococcus, Enterococcus, Enterobacter* (Korpela et al., 2018) and finally *Bifidobacterium* genera, respectively (Korpela et al., 2018; Tauchi et al., 2019).

In preterm infants, patterns of BGM development differ between infants who develop NEC compared to healthy controls: with a higher abundance of *Clostridia* (mainly *Clostridioides sensu stricto*) among infants developing early-onset NEC (NEC onset <23 days of life), and of Gammaproteobacteria (*E. coli* and *Shigella*) in those developing late-onset NEC; in both groups, changes were observed beginning 6 days prior to NEC onset (Zhou et al., 2015). These changes reflect the absence of one common gut bacterial pattern associated with NEC, which varies according to the age of infants developing NEC. In another study, BGM in preterm infants developing NEC tended to be less diverse from days 17–22 postpartum, with a higher abundance of *E. coli;* while metagenomic analysis showed that detection of uropathogenic *E. coli* was a risk factor for both NEC and mortality (Ward et al., 2016). In cohorts of low birth weight infants (<1,500 g) followed by Warner et al. (2016) differences in BGM composition between children with NEC and controls were observed after 30 days postpartum, with increased proportions of Gammaproteobacteria and lower proportions of Negativicutes and the class Clostridia. Yet another study, reported similar overall diversity between preterm infants over 28 weeks of gestational age with NEC and preterm controls, albeit specific differences were observed with increased abundance of *Propionibacterium* among infants with NEC, while *Lactobacillus, Phascolarctobacterium*, and *Streptococcus salivarius* were more abundant in controls; in addition, functionality inference analysis showed that NEC cases had lower xenobiotic biodegradation and metabolic activity compared to controls, suggesting not only an altered composition pattern but also a functionally altered microbiota (Feng J. et al., 2019). In these infants, reduced xenobiotic detoxification by BGM may be related to an inflammatory response in the gut, as described in inflammatory bowel disease (Langmann et al., 2004). Modifications of the microbiota composition are observed not only in advanced NEC stages (Bell’s grades II–III), as in the above studies, but also in grade I stage with changes in the microbiota composition varying according to days of life: e.g., increased abundance of *Streptococcus* during the second 10-days of life, and *Staphylococcus* during the third 10-days of life compared to controls, and of *Raoultella* in NEC stage I cases during the second month of life (Brehin et al., 2020).

Altogether, current evidence suggests that the BGM composition is altered in preterm infants developing NEC compared to preterm controls, characterized largely by a less diverse microbiota, enrichment in certain components, especially Gammaproteobacteria, with a potential influence of specific bacterial components (uropathogenic *E. coli*) in disease outcome. Gram-negative bacteria in mouse models of hypoxia-induced NEC are associated with early histological damage (Carlisle et al., 2011), while members of Clostridia and Bifidobacteriales are producers of SCFA, which in physiological amounts are protective against enterocytes injury (Zheng et al., 2020). Thus, the described disbalance of these BGM components in preterm infants could partially explain NEC development. BGM composition in environments such as NICUs is affected by various factors, e.g., by antibiotic use more than by disease states such as NEC or sepsis itself (Wandro et al., 2018). Large, prospective studies are required to clarify the specific timing at which BGM dysbiosis is initiated, in order to potentially modulate the BGM in order to increase protective bacteria or limit expansion of pathogenic bacteria before NEC onset.

### Rotavirus and Norovirus Infections

Diarrheal disease is an important cause of mortality in children under 5 years of age in low-income countries, and an important cause of medical resource utilization in middle-high income countries (Florez et al., 2020). The leading causes of diarrhea in children are viruses (70–90%). Historically RV was the leading cause of viral diarrhea in children, however, following the introduction of RV vaccines, norovirus (NV) has become the primary cause of moderate-severe diarrhea in several of those countries (Hemming et al., 2013; Bucardo et al., 2014; McAtee et al., 2016). Viruses produce diarrhea through several mechanisms, including damage or death of epithelial cells, altered epithelial absorption, intestinal hypermotility by effects on the enteric nervous system (Crawford et al., 2017), and secretion mediated by viral factors, such as the rotavirus non-structural protein NSP4 (Beau et al., 2007). Immune response to enteric viral infections is mediated by both the innate immune system (including PAMPs-PRR interaction, antiviral IFN-I and II responses) (Villena et al., 2016) and adaptive immunity, including humoral responses (both local IgA and systemic IgM/IgG responses) (Blutt et al., 2012) and cellular responses (LTCD8, LTh1, LTh2, and LTreg) (Malm et al., 2019; Schorer et al., 2020); although the role of cellular response has been less characterized. Studies in animal models have provided insights on the interaction of enteric pathogens and the BGM. RV infection altered the BGM in neonatal-mice, with a time-dependent decrease of *Lactobacillus* and an increase in the mucin-degrading bacteria *Bacteroides* and *Akkermansia* associated with an increased glycan availability in the ileum; yet no changes were found in other intestinal segments (Engevik et al., 2020). Glycan degradation by these bacteria decreases RV decoy-binding to mucins, suggesting that changes in BGM promote RV infection by affecting the protective role of mucus (Engevik et al., 2020). A recent study in neonatal germ-free piglets transplanted with a human child’s microbiota reported bidirectional interactions between the BGM and RV infection, e.g., colonized piglets had reduced RV-induced diarrhea and viral shedding compared to germ-free controls, and RV infection caused alterations in the BGM (Kumar et al., 2018). In addition, a recent study showed that spontaneous resistance to RV infection in mouse colonies was determined by specific BGM composition, specifically by segmented filamentous bacteria, which increase epithelial turnover, protecting against infection (Shi et al., 2019). However, a previous study showed that BGM ablation through antibiotic treatment in mice delayed RV infection and reduced infectivity in mice, enhancing specific antiRV humoral immunity (Uchiyama et al., 2014). NV infection can also alter the BGM in mice, increasing the Bacteroidetes/Firmicutes ratio (Hickman et al., 2014), although this finding has not been consistent (Nelson et al., 2013). On the other hand, antibiotic-induced gut dysbiosis prevents persistent NV infection in mice (Baldridge et al., 2015), which suggests a crucial role of the BGM in promoting NV infection.

The above findings lead to the conclusion that the BGM has bidirectional interactions with RV and NV infections, where BGM composition can either protect against or predispose the host to infection; an infection which in turn can alter the BGM.

While several studies have evaluated BGM composition during acute diarrhea in children (Supplementary Tables 1, 2), there is limited evidence for BGM during diarrheal disease caused specifically by RV and NV in children. A reduction of BGM α-diversity, considered broadly as an “unhealthy microbiota,” was observed in children suffering from RV and NV diarrhea episodes compared to healthy controls (Chen et al., 2017; Xiong et al., 2021); this reduction was more prominent in RV-caused diarrheas (Chen et al., 2017). However, an increase in bacterial abundance (Chao1 index) was found in NV-caused diarrheas compared to healthy controls (Xiong et al., 2021). This is concordant with studies in animal models, where NV requires the presence of determined components of BGM to produce infection, while dysbiosis (e.g., loss of diversity) predisposes the host to RV infection in most studies. Cohort studies in children are needed to assess if these differences in the BGM under stable conditions, differentially predispose the host to either RV or NV infection, and whether these BGM variations persist during diarrhea episodes as observed in cross-sectional studies. RV diarrhea episodes tend to be more severe than those caused by NV (Chen et al., 2017; Xiong et al., 2021), which has also been correlated with decreased α -diversity compared to mild diarrhea episodes (Chen et al., 2017). Beyond diversity, BGM composition is consistently altered during episodes of RV and NV diarrhea compared to healthy controls, but which specific taxa are increased or decreased varies among studies (details in Tables 4, 5). RV-caused diarrhea episodes tend to have a predominance of *Bifidobacterium, Streptococcus, Enterococcus*, and *Lactobacillus* at the genus level compared to healthy controls, and NV-caused diarrhea episodes have a predominance of *Streptococcus* and *Enterococcus* compared to healthy controls (Xiong et al., 2021). *Enterococcus* can bind to NV *in vitro* and predispose the host to infection (Almand et al., 2017); thus the predominance of this taxa during NV-caused diarrhea may indicate a previous predisposition to infection. Interestingly, *Bifidobacterium* and *Lactobacillus* are widely used as probiotics to restore BGM composition, reduce the duration of diarrhea episodes, and to promote an antiviral immune response (Lee et al., 2015); an increase in these “beneficial” bacteria may facilitate restoration of gut homeostasis in RV-induced diarrhea. In linear discriminant effect-size analysis [which aims to determine those taxa that explain the differences between microbial communities (Segata et al., 2011)] – *Bacillus* spp. was the most characteristic taxa in both RV and NV-caused diarrheas compared to healthy controls (Xiong et al., 2021). *Bacillus* spp. are spore-forming bacteria with probiotic properties which reduce duration of diarrhea in children (Ianiro et al., 2018). In a cellular model of RV infection, a mix of *Bacillus clausii* strains prevented RV-induced epithelial cell barrier disruption and inhibited the expression of proinflammatory cytokines including IFN-Beta (Paparo et al., 2020). In murine NV infection, the use of Poly-gamma-glutamic acid (γ-PGA) – an extracellular polypeptide produced by *Bacillus* species- induced a systemic and intestinal IFN-beta response without inducing other proinflammatory cytokines, which was associated with later prevention of NV infection (Lee et al., 2018). Thus, the enrichment of this genus during diarrhea episodes in children may reflect a protective self-regulatory response, if this impacts on immune response and clinical outcome, or if there are different effects on RV and NV- caused diarrheas remains unknown.

**TABLE 4:** Abundance of the gut microbiota’s main phyla and their components during diarrhea episodes compared to healthy controls.

	Description	References
Proteobacteria abundance	− Higher relative abundance of the phylum Proteobacteria and/or its subtaxas (mainly *Escherichia*) compared to healthy controls.	Pop et al., 2014; Braun et al., 2017; Castano-Rodriguez et al., 2018; Kieser et al., 2018; The et al., 2018; Mizutani et al., 2021
	− Infants with viral diarrhea (RV or NV) had lower relative abundance of Proteobacteria compared to healthy children in one study.	Xiong et al., 2021
	− *Escherichia coli* was diminished in diarrheal samples compared to controls in one study.	Samb-Ba et al., 2014
Firmicutes abundance	Higher abundance in healthy controls compared to children with diarrhea, especially those specific components considered markers of a healthy gut microbiota: • Families *Lachnospiraceae*, *Ruminococcaceae* and *Erysipelotrichaceae.* • Members of the *Clostridiaceae* family: *Faecalibacterium*, and other specific *Clostridiales*.	Pop et al., 2014; Chen et al., 2017; Gallardo et al., 2017; Gigliucci et al., 2018; Kieser et al., 2018; The et al., 2018; Mizutani et al., 2021
	− Bacterial genera of the *Lactobacillales order such as Streptococcus* were abundant in infectious diarrheas irrespective of their etiology, and in STEC-associated diarrheas. − *Streptococcus* and *Enterococcus* had higher abundance in RV and NV diarrhea compared to healthy controls.	Pop et al., 2014; Becker-Dreps et al., 2015; Gigliucci et al., 2018; Kieser et al., 2018; The et al., 2018; Mathew et al., 2019; Xiong et al., 2021
	− A *Faecalibacterium*-predominant enterotype in diarrhea episodes occurring in children and adults has been described	Castano-Rodriguez et al., 2018
Bacteroides abundance	− Bacteroidota were increased in diarrheal samples of children irrespective of their etiology, and in children with diarrhea caused by diarrheagenic *Escherichia coli* (DEC) compared to healthy controls	Becker-Dreps et al., 2015; Gallardo et al., 2017, 2020; Castano-Rodriguez et al., 2018
	− The genus *Bacteroides* constituted a specific enterotype in children from Vietnam with diarrhea irrespective of etiology; while a diminished abundance was observed in children and adults from Bangladesh and in children from Senegal and China with RV-diarrhea compared to controls.	Samb-Ba et al., 2014; Chen et al., 2017; Kieser et al., 2018; The et al., 2018
	− The genus *Parabacteroides* was reduced in RV-diarrhea compared to controls	Chen et al., 2017
	− The genus *Prevotella* was increased in RV-bacterial co-infections, and in children from Chile with DEC, but was diminished in children with diarrhea from Bangladesh and South Africa irrespective of etiology compared to controls.	Pop et al., 2014; Kieser et al., 2018; Mathew et al., 2019; Gallardo et al., 2020

**TABLE 5:** Differences in the gut microbiota composition during childhood infectious diarrhea according to etiology.

	Description	References
Bacterial v/s viral diarrheas	In children from Bangladesh, the abundance of *Escherichia coli* was increased compared to controls, except in patients with RV infection compared to bacterial pathogens (DEC, *Aeromonas, Vibrio, Campylobacter*, and *Shigella*); other viruses were not analyzed.	Kieser et al., 2018
	In children from Vietnam with diarrhea, when cluster analysis based on β -diversity data was performed, samples were segregated into 4 community-structure types (or Enterotypes). The *Streptococcus*-dominant enterotype was more common in samples with a bacterial pathogen detected (*Campylobacter, Salmonella*, and *Shigella*) compared to the *Bifidobacterium*-dominant, *Bacteroides*-dominant, and *Escherichia*-dominant enterotypes.	The et al., 2018
	In children from Chile, DEC infection was associated with a higher proportion of Proteobacteria and a lower proportion of Firmicutes at the phylum level, a greater abundance of *Enterobacteriaceae* at the family level, and a greater abundance of the genus *Escherichia-Shigella* compared to viral infections (RV, NV, Adenovirus, Astrovirus, and Sapovirus).	Gallardo et al., 2017
Viral v/s virus-bacteria co-infection	In children from Qatar with RV or NV-diarrhea, co-infection with bacteria was associated with differences in microbiota composition: RV co-infection with EAEC was associated with a predominance of *Streptococcus* and *Escherichia*, while co-infection with EAEC and EPEC was associated with an abundance of *Prevotella* and *Escherichia*; NV co-infection with EAEC and EPEC was associated with a dominance of *Streptococcus*, *Escherichia*, and *Clostridium* genera, and a lower abundance of *Enterococcus* and *Veillonellaceae* compared to viral-only infections.	Mathew et al., 2019
Different viral pathogens	In children from Chile with viral diarrhea, microbiota composition of samples with different enteric viruses clustered together.	Gallardo et al., 2017
	In children from Taiwan with viral diarrhea, children with RV infection had a significantly lower α-diversity score compared to children with NV, and the latter was no different from controls.	Chen et al., 2017
	In children from China with viral diarrhea, the RV group had lower α-diversity (Simpson index) than the NV group. The RV group exhibited higher abundances of Actinobacteria and Verrucomicrobia at the phylum level, and higher abundances of *Veillonella* and *Bifidobacterium* at the genus level compared to NV group; the NV group had a higher abundance of Fusobacteria at the phylum level, and *Enterococcus*, *Clostridium*, and *Fusobacterium* at the genus level compared to the RV group.	Xiong et al., 2021
Different bacterial pathogens	In a group of adults and children with bacterial infections (*Campylobacter, Salmonella, Shigella*, and STEC) microbiota composition did not vary significantly between different etiologies. In children from Chile with DEC-associated diarrhea, when a community structure analysis was performed, microbiota composition of samples with different DEC pathotypes were clustered in independent groups	Singh et al., 2015; Gallardo et al., 2017

Clinical manifestations related to viral diarrhea are also associated with specific BGM taxa, e.g., abdominal pain was related to the abundance of *Prevotella* (Chen et al., 2017), which is associated with a proinflammatory response in several chronic diseases (Larsen, 2017), but also to a lack of effective response to the RV vaccine (Harris et al., 2017a); convulsions were related to a substantial decrease in *Faecalibacterium* (Chen et al., 2017), a SCFA producer important in the maintenance of regulatory intestinal immunity (Alameddine et al., 2019). Whether specific BGM components influence clinical outcomes in viral diarrhea episodes through immune mechanisms, as postulated here, remains to be clarified. Functional predictions of the BGM in RV and NV diarrhea episodes showed an increase in chloroplast and photosynthesis pathways compared to healthy controls, which may be explained by incomplete BGM-dependant digestion of plant food in the gut during diarrhea (Xiong et al., 2021).

### Enteric Bacterial Infections With a Focus on Diarrheagenic *E. coli*

Bacteria cause approximately 10–20% of diarrhea episodes in children, of which *Shigella, Salmonella, Campylobacter*, and enterotoxigenic *E. coli* (ETEC) predominate; less common causes include enteroinvasive *E. coli* (EIEC) (Florez et al., 2020). Shiga-toxin producing *E. coli* (STEC) is particularly important in children, as it is the most common cause of hemolytic uremic syndrome (HUS) (Exeni et al., 2018). Bacteria produce diarrhea through both common and specific mechanisms: inflammation occurs during infections with cytotoxin-producing bacteria (*Shigella* and STEC) and in the case of bacteria that invade and disrupt intestinal mucosa (*Salmonella*, *Campylobacter*). This leads to inflammation and necrosis of the epithelium and sub epithelium microabscesses (Florez et al., 2020). Secretory diarrhea occurs when bacteria producing toxins increase intracellular cAMP or cGMP levels (e.g., *Vibrio cholerae* and ETEC) (Thiagarajah et al., 2015). Interaction between the BGM and bacterial enteric pathogens has been evaluated in animal models, with *Citrobacter rodentium* infection widely used to mimic human diarrheagenic *E. coli* in mice. *C. rodentium* induces time-dependant changes in BGM with a rapid decrease in colonic *Mucispirillum* during the early phases, and increases in members of the Clostridiales and Lactobacillales families followed by successful resolution of colitis (Belzer et al., 2014). On the other hand, the BGM (butyrate-producing bacteria) appear to be essential in protecting against *C. rodentium* infection (Wlodarska et al., 2011; Osbelt et al., 2020), again demonstrating the bidirectional interaction between enteric bacterial pathogens and the BGM.

In children with DEC-caused diarrhea, changes in BGM composition during bacterial pathogen infections are characterized by an increase in Proteobacteria and a decrease in Firmicutes (Gallardo et al., 2017), also observed in other bacterial pathogens in children and adults along with a decrease in α-diversity (Singh et al., 2015). Increases in Proteobacteria can be partially explained by an increase in *Escherichia/Shigella* species as the cause of diarrhea, but also by other members of *Enterobacteriaceae*, such as *Citrobacter* and *Enterobacter* (Gallardo et al., 2017). These changes in BGM composition are associated with specific metabolic functions, as DEC samples display enrichment in pathways involved in histidine degradation, while healthy controls were enriched in L-ornithine and L-histidine biosynthesis pathways, correlating with higher levels of histamine (an histidine-degradation product) and lower levels of ornithine, explained mainly by the presence of *Enterobacter hormaechei*, *Citrobacter werkmanii/freundii, Shigella* spp., and *Bifidobacterium stercoris* (Gallardo et al., 2020). Histamine production is induced by proinflammatory environments and associated with *E. coli* adherence, while ornithine is associated with a healthy microbiota and the maintenance of an unscathed intestinal barrier; these findings suggest that microbiota metabolites may be related to DEC pathogenesis (Gallardo et al., 2020). Whether this determines the clinical impact of infection needs to be explored further. The decrease in Firmicutes during STEC-caused diarrhea episodes is primarily explained by decreases in Clostridiales (including the genera *Lachnospiraceae* and *Ruminococcaceae*) compared to healthy controls (Gigliucci et al., 2018). This occurs alongside a decrease in Bifidobacteriales, all recognized as SCFA producers, metabolites which can modulate the expression of flagellin, chemotaxis proteins and adhesins either promoting or limiting pathogenicity depending on SCFA concentrations in the intestinal lumen (Tobe et al., 2011; Navarro-Garcia, 2014; Lackraj et al., 2016). Whether the reduction of these bacteria during STEC-diarrhea is accompanied by changes in SCFA levels, and how this may influence the immune response and clinical outcomes in children remains undetermined.

In comparison to viral only infections, co-infection with DEC is associated with higher clinical severity scores (Mathew et al., 2019) and with changes in BGM composition, which is dependant on the DEC pathotype, e.g., RV + EAEC co-infection is associated with higher *Streptococcus* abundance, which is not observed in RV + EPEC co-infections. Interestingly, an increase of the potentially beneficial *Bifidobacterium*, previously described in viral diarrheas, is more marked in virus-DEC co-infections (Mathew et al., 2019) which have higher clinical severity scores. This indicates that BGM changes during diarrhea episodes are variable between studies, and it is important to assess BGM composition separately in different diarrheal etiologies, as the role of the BGM during acute diarrhea seems to be pathogen-dependent.

**FIGURE 1:**
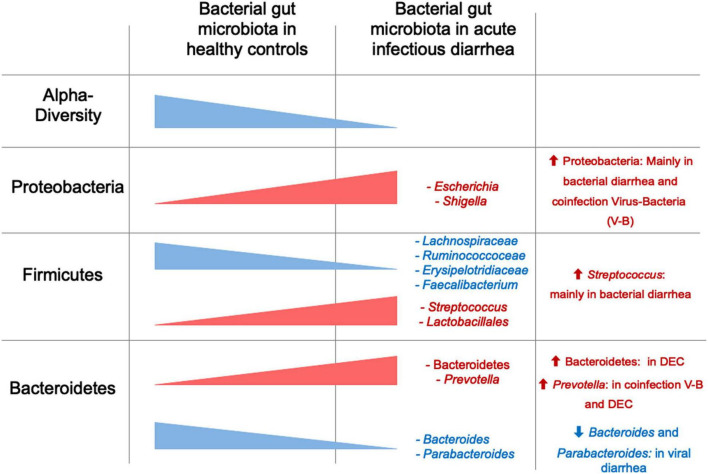
α-diversity and relative abundance of main taxa in children with acute diarrhea compared to healthy controls. Red represents higher diversity or abundance in children with diarrhea, and blue represents higher values in healthy controls.

Thus far, we have summarized studies in children focused on specific pathogens. In Supplementary Table 2 we describe studies that do not determine diarrhea etiology, and/or do not clearly separate their analysis according to etiology or age groups. Considering the global data, we can conclude that the BGM composition is altered during episodes of infectious diarrhea in children, characterized by decreased α-diversity and modifications in predominant bacteria, with an increase in Proteobacteria (mainly *Escherichia*) and a decrease in Firmicutes, while changes in Bacteroides and other groups are variable (Figure 1 and Table 4). In addition, the etiologic agent is associated with differences in BGM composition, e.g., bacterial only vs. virus-bacteria co-infections tend to display different microbiota patterns compared to those caused by viruses in the absence of bacteria (Table 5). Overall microbiota recovery toward a “healthy-status microbiota” begins several weeks after diarrhea resolution (Becker-Dreps et al., 2015; Singh et al., 2015), which is influenced by the etiologic agent and other potential but less explored factors, such as diarrhea severity, social context, and age.

### *Clostridioides difficile* Infection

*Clostridioides difficile* is present in the gut microbiota of around 30% of healthy infants <1 month of age; this decreases to approximately 10% at 12 months and 0–3% at 3 years of age (Jangi and Lamont, 2010). Why infants with CD in their BGM do not develop disease is unknown. Studies in newborn rabbits suggest that the lack of toxin receptors on the surface of epithelial cells may play a role (Eglow et al., 1992). In certain situations CD can cause mild to severe disease, especially in children suffering a malignancy, recent surgery, antibiotic exposure, or solid organ transplantation (Nicholson et al., 2015; Anjewierden et al., 2019). These situations may lead to characteristic disruptions in the BGM and the absence of an efficient immune response to CD-infection (Buonomo and Petri, 2016). The pathogenicity of CD is attributed to its production of the toxins A (enterotoxin) and B (cytotoxin), which inactivate GTP-binding proteins, causing cytoskeleton disruption and apoptosis of colonic cells (Abt et al., 2016). Disruption of the epithelial barrier causes bacterial translocation from the gut and access to other tissues. This disruption and translocation activates an inflammatory response (pro-inflammatory cytokines, chemokines, recruitment of innate and adaptive immune cells, reactive oxygen species production) that can limit the systemic spread of CD (Abt et al., 2016). However, an exaggerated inflammatory response can be detrimental in exacerbating epithelial damage and increasing the severity and duration of disease, as shown in mouse models (Buonomo et al., 2013). Animal models of CD infection have been constructed dominantly of mice treated with antibiotics, which causes gut dysbiosis characterized by a decrease in richness, a decrease in Bacteroidetes and an increase in Proteobacteria or *Enterobacteriaceae* (Theriot and Young, 2015), with specific differences between studies possibly related to the administration of different antibiotics. These changes in the BGM are related to susceptibility to CD-caused disease and the restoration of this microbiota using specific strains e.g., a mix of *Staphylococcus warneri*, *Enterococcus hirae*, *Lactobacillus reuteri*, *Anaerostipes* sp. nov., *Bacteroidetes* sp. and *Enterorhabdus* sp. resolves CD-recurrent disease in mice (Lawley et al., 2012).

CD-associated diarrhea (CDAD) is uncommon in children, especially during the first years of life, and only a few studies have analyzed BGM composition during CD infection in this age group. In children from China with CDAD, α-diversity was decreased compared to healthy controls. In these children, differences in microbiota composition at the phylum level (higher abundance of Firmicutes, Actinobacteria, Proteobacteria and Acidobacteria, and lower abundance of Bacteroidetes), as well as the genera level (increase in *Enterococcus*, *Streptococcus*, *Escherichia/Shigella, Klebsiella*, *Stenotrophomonas* and *Haemophilus*, and decrease in *Bacteroides, Faecalibacterium, Parabacteroides, Lachnospiracea incertae sedis, Dialister*, and *Alistipes*) were observed when compared to healthy controls (Ling et al., 2014). In hospitalized children from the United Kingdom with acute diarrhea, CD carriage (detected by culture and lateral flow test) was associated with a higher α-diversity index, and a higher abundance of the families Lachnospiraceae and Ruminococcaceae compared to culture-negative children (Lees et al., 2020). These findings are contrary to those described by Ling et al. (2014) in Chinese children; in this study detection of CD was associated with a decrease in the abundance of butyrate-producing bacteria from the *Ruminococcaceae* and *Lachnospiraceae* families. This coincides with results from adults with CD-associated diarrhea (Antharam et al., 2013). A protective role of butyrate in preventing CD disease has been described in animal models, given that antibiotics that predispose the host to CD-infection deplete butyrate levels (Theriot et al., 2014), and butyrate reduces intestinal epithelial permeability, inflammation and bacterial translocation in infected mice (Fachi et al., 2019). The potential role of differences in the abundance of BGM butyrate-producing bacteria in relation to different clinical outcomes has not been characterized; metabolomic studies in children with CD-associated diarrhea would be useful in determining the validity of this hypothesis.

The toxigenic status of CD also determines differences in microbiota composition. In children from China with CD-associated diarrhea, α-diversity was lower in those with CD producing both toxins A and B (A+B+) compared to those producing only toxin B (A−B+); CD A+B+ was associated with increased abundance of Firmicutes and Acidobacteria at phylum level, increased abundance of *Lactobacillales* at order level and *Enterococcaceae* at the family level compared to CD A−B+ (Ling et al., 2014). In the cohort from the United Kingdom, the presence of toxigenic CD was associated with an increased abundance of Proteobacteria and a decreased abundance of Firmicutes and Bacteroidota at the phylum level, and an increased abundance of *Klebsiella* at the genus level compared to non-toxigenic CD carriers and culture-negative children (Lees et al., 2020). Differences in both studies may be partially explained by the age of children studied, as Chinese children ranged from 2 to 4 years of age while children from United Kingdom ranged from 0 to 16 years of age.

In animal models, metabolism of bile acids driven by the BGM has a protective role by inhibiting spore germination and overgrowth of CD-vegetative cells (Theriot et al., 2014); whether this (or other) mechanisms explain why some children are asymptomatic carriers of inactive forms of CD, while others are toxin-producers and can develop CD-associated disease is still unknown.

The fact that fecal transplantation, which aims to restore a “healthy gut microbiota,” has proven effective in the treatment of recurrent CD in adults and children, supports that gut microbiota disruption is a key factor in the development of CD-disease. In children, fecal transplantation causes an increase in α-diversity (which was particularly reduced in children requiring multiple transplants to achieve clinical success), a decrease in Proteobacteria and an increase in Bacteroidota, evolving toward a “healthy microbiota” in parallel with clinical improvement (Fareed et al., 2018; Li X. et al., 2018). Metabolite-mediated mechanisms related to BGM restoration have been described in FMT (Martinez-Gili et al., 2020). Bile salt hydrolases (BSH)-producing bacteria present in adult donor samples have been associated with a decrease of taurocholic acid (TCA) in recipient stools, a conjugated bile acid that triggers CD germination (Mullish et al., 2019). Also, a restoration of SCFA levels is seen in FMT adult recipients (Seekatz et al., 2018), which might be partially explained by changes in diet after FMT or antibiotic cessation post resolution of recurrent CD-disease as discussed by other authors (Martinez-Gili et al., 2020). In an *in vitro* model of CD infection, the SCFAs succinate, butyrate, acetate, and isobutyrate, decrease when antibiotics are discontinued, and are not affected by a later FMT. However, recovery of valerate levels is only seen after FMT, which is related to inhibition of CD growth (McDonald et al., 2018). As we previously described, SCFA have anti-inflammatory effects which can participate in CD-recovery after FMT by other mechanisms beyond inhibition of CD growth. It is important to consider that FMT is associated with changes in gut virome and mycobiome (Fujimoto, Zhang), which is not intended to be discussed in this review, but highlights the fact that even when BGM disruption seems to be a key factor in CD-disease, the FMT effects are multifactorial and most likely only partially mediated by the restoration of BGM and bacterial metabolites.

## Gut Microbiota and Respiratory Infections During Early Childhood

Acute respiratory infections (ARIs) are a major source of morbidity and mortality in children worldwide (United Nations Inter-agency Group for Child Mortality Estimation, 2019), representing the second leading cause of years of life lost due to premature mortality and one of the most frequent causes of hospitalization (Nair et al., 2013). These infections can range from mild upper ARIs (AURIs), such as the common cold, to life-threatening conditions including lower ARIs (ALRIs), such as pneumonia and bronchiolitis. ARIs are the most frequent type of infection during the first 3 years of life, with a median of 10 episodes per child (with up to 90% corresponding to AURIs) (Vissing et al., 2018). Viruses are the primary cause of both AURIs and ALRIs in children, with rhinovirus the most common cause of AURI (Kwiyolecha et al., 2020) and respiratory syncytial virus the most common cause of ALRI (O’Brien et al., 2019; Li et al., 2021). Influenza virus causes seasonal global epidemics, representing an important cause of ALRIs associated with hospitalization and severe outcomes in children aged <5 years as compared to older children and adults (Ruf and Knuf, 2014; Wang et al., 2020). Adenovirus, parainfluenza, metapneumovirus, and enterovirus (among others) also cause ARIs in children, however, they are less common (Benet et al., 2017; Chen J. et al., 2018). Bacteria are also a major cause of ALRIs, with *Streptococcus pneumoniae* causing the majority of infections despite global vaccination efforts, resulting in significant morbi-mortality in children <5 years of age (Wahl et al., 2018).

The RSV pathogenesis has been studied thoroughly. The virus replicates in the airway epithelium, which can result in lower airway inflammation, alveolar epithelial cell apoptosis, bronchial epithelial necrosis, multifocal acute alveolitis, intra-alveolar edema, and hemorrhage. An intense neutrophil response during this early phase is positively correlated with tissue damage and disease severity (Russell et al., 2017; Sebina and Phipps, 2020). Activation of adaptive immunity characterized by cytotoxic cells and Th1 cells are protective, mediating viral clearance; Th2-skewed responses on the other hand appear to be deleterious and are associated with severe disease outcome (Caballero et al., 2015) and may be related to a future risk of asthma development (Restori et al., 2018).

### Linking the Gut Microbiota and Respiratory Infections: The Gut-Lung Axis

There is extensive evidence that the BGM may influence immunity at the respiratory tract level, resulting in protection, predisposition or modification of respiratory infection/disease. The mesenteric lymphatic system can translocate intact bacteria, fragments, or metabolites from the intestinal lumen to systemic circulation, reaching the respiratory tract and modulating the immune response at this level (Enaud et al., 2020). The gut-lung axis has been extensively reviewed by other authors (Budden et al., 2017; Dumas et al., 2018; Enaud et al., 2020; Sencio et al., 2021). We summarize the main components mediating BGM modulation over lung immunity in Table 6.

**TABLE 6:** Key factors involved in the modulation of lung immunity through the Gut-Lung axis.

Gut microbiota component	Description	Interaction with respiratory tract immunity	Effect on lung immunity	References
SCFA	Metabolites derived from gut microbiota fermentation of undigested dietary fibers. Propionate, acetate, and butyrate are the main SCFA.	− SCFA translocate from gut to the systemic circulation and reach the bone marrow, where they promote hematopoiesis and differentiation of different lineages depending on context (e.g., during influenza infection they induce monocytes and dendritic cell progenitors differentiation and increase of patrolling macrophages which reach the lung).	↑ or ↓	Arpaia et al., 2013; Trompette et al., 2014; Antunes et al., 2019
		− Acetate promotes a type I-IFN response in pulmonary epithelial cells, in a Gpr43 receptor-dependent manner.		
		− During influenza infection, SCFAs have a direct effect on LTCD8 activation by enhancing cellular metabolism in a GPR41 (G protein coupled receptor) dependent manner.		
		− SCFAs promote an extrathymic peripheral Treg cell pool, associated with decreased allergic airway diseases through histone deacetylase inhibition.		
Segmented filamentous bacteria (SFB)	Commensal gut microbiota belonging to the Firmicutes phylum; colonization in humans occurs during the first 2 years of life, with an important decrease after 36 months.	− SFBs promote differentiation of TCD4 to Th17 in a IL1R-dependent manner during pulmonary fungal infections in mice.	↑	Yin et al., 2013; Chen B. et al., 2018
		− Th17 response induced by SFB is involved in lung autoimmune disease in mice.		
Desaminotyrosine	Degradation product of flavonoids, plant-derived polyphenol compounds with intestinal and systemic anti-inflammatory effects.	− Desaminotyrosine produced by *Clostridium orbiscindens in the gut* promotes a pulmonary type 1 IFN response, protective against Influenza infection.	↑	Steed et al., 2017; Wei et al., 2020
PRR agonists	Activation of PRR, including Toll-like receptors (TLR) and Nod-like receptor (NLR) by gut-bacterial ligands enhance antiviral respiratory immune responses.	−Gut TLR4 activation by LPS induces protection against *E. coli* pneumonia in mice by promoting a NFKB response and activation of alveolar macrophages.	↑	Chen et al., 2011; Ichinohe et al., 2011; Clarke, 2014
		− Gut (NLR) activation by their ligands determines an effective lung immunity to *Klebsiella pneumoniae* by promoting alveolar macrophage ROS-mediated bacterial killing.		
		− Rectal inoculation of TLR agonists CpG (TLR9 agonist), Poly I:C (TLR3 agonist), peptidoglycan (TLR2 agonist), and LPS (TLR-4 agonist) restore immunity to influenza virus in antibiotic-treated Mice. TLR-5 activation by Flagellin is necessary for antibody responses against influenza virus vaccination in mice.		

### Gut Microbiota During Acute Respiratory Infections in Children and Mouse Models

Lung infections can induce changes in the gut microbiota, establishing a bidirectional axis between the respiratory tract and the gut microbiota.

Four microbiota profiles were identified among children hospitalized with bronchiolitis (65% with RSV and 23% with Rhinovirus) and healthy controls, e.g., *Escherichia*-dominant, *Bifidobacterium*-dominant, *Enterobacter/Veillonella*-dominant, and *Bacteroides*-dominant. The proportion of children with bronchiolitis was significantly higher in the Bacteroides-dominant profile; these children also had increased bacterial richness and α-diversity. The *Enterobacter/Veillonella*-dominant profile had the lowest proportion of children with bronchiolitis (Hasegawa et al., 2017). In addition, functional predictions based on taxonomy indicated that the BGM of children with bronchiolitis had increased abundance of gene functions related to sphingolipid metabolic pathways compared to controls, which may be associated with an immunomodulatory response (Hasegawa et al., 2017). In hospitalized children with RSV bronchiolitis, diminished α-diversity was observed in the BGM compared to healthy controls; additionally, alterations in composition were observed, characterized by enrichment in Proteobacteria and Bacteroidota at the phylum level and enrichment in *Bacteroides* and *Streptococcus* at the genus level, as compared to healthy children. Conversely, the oropharyngeal microbiota in children with bronchiolitis was enriched in Firmicutes and depleted in Bacteroidota and Proteobacteria compared to healthy controls. Both the fecal and oropharyngeal microbiota remained relatively unchanged 7–10 days after admission (Hu et al., 2017). The quantity of *Bifidobacterium* was decreased in fecal samples of wheezing children hospitalized with bronchiolitis or asthma of unknown etiology, which was in turn associated with lower serum levels of Th1 cytokines and higher levels of serum Th2 and Th17 cytokines, as compared to controls (Liwen et al., 2018).

In summary, relatively limited data suggests that the BGM is altered in children with bronchiolitis or wheezing episodes when compared to healthy age-matched controls, with a reduction in α-diversity, an increase in the phylum Bacteroidota (possibly associated with specific sphingolipid metabolic pathways) and a reduction in *Bifidobacterium* (possibly associated with serum Th2/Th1 imbalance). As these studies are cross-sectional, the question as to whether these alterations in the BGM are the result of or the cause of the host’s predisposition to ARIs is unclear. The latter theory is supported by a recent birth-cohort study from The Netherlands aimed at assessing the impact of delivery mode on the gut microbiota and health during the first year of life. This study concluded that there is an association between microbiota composition during the first week of life and the number of ARIs later in life; specifically the presence of *Bifidobacterium* was associated with fewer ARIs and the presence of *Klebsiella* and *Enterococcus* with more ARIs over the first year (Reyman et al., 2019). The fact that a “healthy” BGM has protective effects in the case of ARIs is also supported by studies in mouse models, e.g., the transfer of a “healthy” BGM from wild mice to laboratory mice resulted in reduced inflammation and increased survival following influenza infection (Rosshart et al., 2017); furthermore, gut microbiota-derived SCFAs (acetate) protected mice against RSV infection, through the promotion of type I IFN in pulmonary epithelial cells (Antunes et al., 2019). Conversely, experimental RSV and influenza infections in mice caused an alteration in their gut microbiota, with an increase in Bacteroidota (mainly due to *Bacteroidaceae*) and a decrease in Firmicutes (mainly *Lachnospiraceae* and *Lactobacillaceae* families) (Groves et al., 2018). Also, RSV infection altered lipid metabolism, increasing fecal abundance of sphingolipids, polyunsaturated fatty acids, and the SCFA valerate (Groves et al., 2020), supporting the gut microbiota metabolic functional prediction in children cited before (Hasegawa et al., 2017). Nevertheless, the theoretical impact of these changes on anti-inflammatory responses remains unclear.

For the purpose of this review, we were not able to find publications on the relationship between the BGM and other ARIs such as influenza or pneumococcal pneumonia, but there is rising evidence coming from animal models. Studies in mice focusing exclusively on the effect of viral influenza on the BGM describe an association with dysbiosis, primarily characterized by an increase in Proteobacteria or *Enterobacteriaceae* (Wang et al., 2014; Deriu et al., 2016; Bartley et al., 2017) and a decrease in Bacteroidetes (Yildiz et al., 2018) and/or Firmicutes (Wang et al., 2014). BGM alterations seem to be mediated by the inflammatory response initiated in the respiratory tract and through a systemic release of cytokines, type I-IFN (Deriu et al., 2016) and IFN-gamma (Wang et al., 2014), leading to intestinal imbalances that predispose the host to invasion by bacterial pathogens (Deriu et al., 2016; Yildiz et al., 2018).

A healthy BGM seems protective against *Streptococcus pneumoniae* infection in mice, as antibiotic-induced depletion of the gut microbiota was associated with worse outcomes compared to controls, partially mediated by decreased lung macrophage phagocytosis capacity (Schuijt et al., 2016). Segmented filamentous bacteria protected against pneumococcal pneumonia in immunocompromised mice Rag -/- by promoting neutrophil resolution following lung infection (Felix et al., 2018). Whether these findings have potential correlations in children, remains unclear.

## Discussion and Therapeutic Projections

**FIGURE 2:**
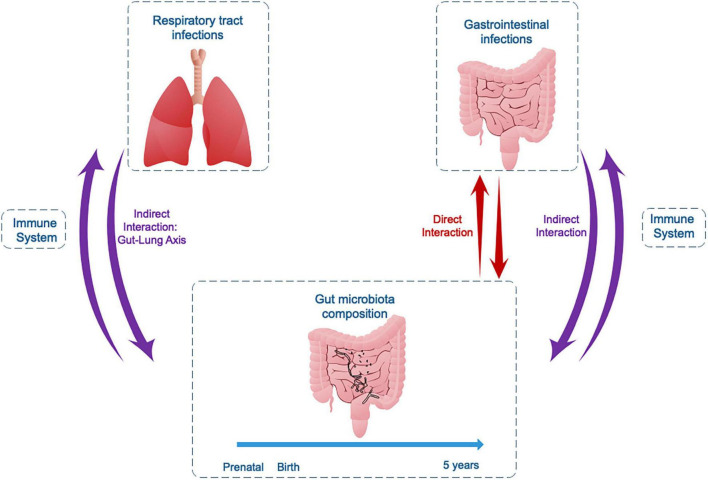
Bidirectional interactions between the gut microbiota and gastrointestinal or respiratory tract infections in childhood.

The development and the dynamics of BGM composition are closely related to the development of the immune system during first years of life, modulating protection or predisposition to infections. Studies in animal models and humans, mainly children, allow us to conclude that bidirectional interactions between infectious microorganisms and the gut BGM are common (Figure 2). One example is the process of gut commensal bacteria development and metabolite production, which interact directly with the offending bacteria or indirectly through immune-stimulated interactions. In the opposite direction is the effect of infection on modifications of the pre-existing BGM, resulting in dysbiosis, which progressively returns to a “healthy” BGM after the pathogen is cleared. Although there are direct interactions between components of the BGM and gastrointestinal pathogens (Table 3), host immune system stimulation seems to be the key mediator as exemplified by the “gut-lung” axis model. This immune modulatory role is further supported by several studies on immune response to vaccines, not reviewed here (Lynn et al., 2021), where the microbiota component is associated with differential immune responses to several vaccines in children in various populations, including the RV, the BCG, and the influenza vaccines.

The specific components of the microbiota, immune system, and infectious agents that play a role in these interactions remains a matter of active research. Studies in children reviewed here have primarily been performed during acute infections, hindering assessment as to whether any differences could be explained by the infection itself, or rather pre-existing dysbiosis. Large cohort and longitudinal studies in healthy children are needed to better understand the intra-individual evolution from a developing healthy gut microbiota to an altered gut microbiota during an infectious episode, including possible convalescence.

Several attempts to modulate or modify the BGM in order to protect against infectious diseases have been explored, of which probiotics have been the most deeply studied. Clinical efficacy of this strategy in reducing the duration and intensity of gastroenteritis symptoms is weak (Szajewska et al., 2020), partially because studies have used diverse probiotic strains. In children with non-specific acute diarrhea, treatment with *Lacticaseibacillus rhamnosus* was associated with modulation of the gut microbiota, characterized by the attenuation of dysbiosis, higher fecal IgA levels, lower intestinal inflammatory markers (lactoferrin and calprotectin), and improved clinical outcomes compared to non-treated children (Lai et al., 2019). In children with acute RV-diarrhea, administration of *S. boulardii* was associated with reduced α-diversity and altered composition (an increase in Proteobacteria and a decrease in Firmicutes) during first 5 days of the diarrhea episode, but not at days 10–30, as compared to healthy controls; although a non-treated group was not evaluated in this study to corroborate if probiotics had a benefit in the recovery of the gut-microbiota (Dinleyici et al., 2018). In children with infectious diarrhea of any cause, administration of *S. boulardii* was associated with the recovery of α- and β-diversity over the 15 days following treatment to levels comparable to healthy controls, which was not observed in non-treated children with diarrhea (Toro Monjaraz et al., 2021). It is noteworthy that the effect of probiotics on BGM composition during diarrhea has been assessed using *S. boulardii*, a fungus closely related to *Saccharomyces cerevisiae*, which is detected in stools of healthy children (Strati et al., 2016). The mechanisms related to this specific probiotic include direct binding to pathogens, induction of antimicrobial peptides and immunomodulatory effects, among others (Pais et al., 2020), similar to mechanisms of interaction between bacterial BGM and pathogens previously discussed in this review. Though mycobiome was not assessed in this article, these findings highlight the complex equilibrium and interactions between different components of the microbiota and the host. For NEC prevention in preterm infants, clinical guidelines recommend specific probiotic strains with little evidence of efficacy (van den Akker et al., 2020). The clinical benefit of certain strains in preterm infants seems to be dependent on their effects on the BGM, e.g., *Bifidobacterium breve* strain BBG-001 did not affect NEC incidence in a multicenter, randomized, controlled phase 3 trial including preterm infants (PiPS trial). There was no difference in microbial richness and diversity of the gut microbiota in the probiotic and placebo groups (Millar et al., 2017). Conversely, a combination of 3 probiotic strains (*Bifidobacterium longum* subsp. *infantis* BB-02, *Streptococcus thermophilus* TH-4, and *Bifidobacterium animalis* subsp. *lactis* BB-12) was associated with a 54% reduction in Bell stage 2 or greater NEC, with significant changes in the gut microbiota compared to placebo (increased in *Bifidobacterium* and decreased in *Enterococcus*) (Plummer et al., 2018). Probiotics have been associated with some protective effect in preventing and reducing the duration of ARIs in children (Wang et al., 2016; Laursen and Hojsak, 2018); but to our knowledge there are no studies simultaneously evaluating their effect on the BGM.

Prebiotics, defined as “substrates selectively utilized by host microorganisms conferring a health benefit” (Gibson et al., 2017), synbiotics, defined as “mixture comprising live microorganisms and substrates selectively utilized by host microorganisms that confers a health benefit” (Swanson et al., 2020), and more recently postbiotics, defined as “preparation of inanimate microorganisms and/or their components that confers a health benefit” (Salminen et al., 2021) are additional potential therapeutic tools that may influence infections by action on the microbiota-immune system axis. A deeper understanding of the associated mechanisms and their clinical relevance in pediatric infectious diseases will be required before advancing these and other preventive or therapeutic interventions. Finally, the use of fecal transplantation to restore a “healthy BGM” beyond CD infection, e.g., for prevention of multi-drug resistant infections in colonized patients has been proposed but unexplored in children (Gurram and Sue, 2019). Whether the use of these therapeutic or preventive BGM-related tools during certain periods of early childhood within a “window of opportunity” can significantly affect the predisposition to, or clinical outcomes of infections during adolescence and adulthood is still not completely understood and requires large cohort studies.

This review has focused on the role of the bacterial gut microbiota on the most common infections in childhood (acute diarrhea and acute respiratory infections) and characteristics of infection-related gastrointestinal entities (necrotizing enterocolitis and *C. difficile* infection). However, several aspects of the interaction between the BGM and infections have not been explored in depth, including the influence of the BGM on vaccine response (Lynn et al., 2021) and its role in antimicrobial efficacy, acting as reservoirs for AMR genes (Anthony et al., 2021) or even modulating the metabolism and pharmacokinetics of antimicrobials (Zimmermann et al., 2019; Klünemann et al., 2021). Finally, there is increasing evidence about the importance of viruses, fungus, archaea and parasites in maintaining the gut homeostasis which was not included in this review. The role of these microorganisms in dysbiosis during common childhood infections, and how variations in these components affect the bacterial GM composition are matters to be assessed.

## Author Contributions

SG and MO’R conceived the original idea. SG and XA performed literature search and study selection. SG and MO’R wrote the manuscript with substantial support from XA, YL, JT, PG, MF, and RV. All authors approved the final manuscript.

## Conflict of Interest

The authors declare that the research was conducted in the absence of any commercial or financial relationships that could be construed as a potential conflict of interest.

## Publisher’s Note

All claims expressed in this article are solely those of the authors and do not necessarily represent those of their affiliated organizations, or those of the publisher, the editors and the reviewers. Any product that may be evaluated in this article, or claim that may be made by its manufacturer, is not guaranteed or endorsed by the publisher.
