# Supplementary material for: Bacterial Gut Microbiota and Infections During Early Childhood
Source: Front Microbiol. 2022 Jan 5;12:793050. doi: 10.3389/fmicb.2021.793050 (PMC8767011; doi:10.3389/fmicb.2021.793050)
Supplement: Supplementary file 1 [file Table_1.docx]

| **Supplementary Table 1: Studies assessing the bacterial gut microbiome composition during acute diarrhea episodes in children caused by specific agents** | | | | |
| --- | --- | --- | --- | --- |
| **Reference, country** | **Country** | **Study design, included patients** | **Etiology of diarrhea: diagnostic methods and detection rates** | **Results** |
| Gallardo et al. Front. Cell. Infect. Microbiol. 2017. | Chile | Fecal microbiome composition of diarrheal samples from children 1-5 years of age (N=63) and healthy controls (N=30) | FilmArray® GI Panel   - DEC as sole pathogen= 32 (EAEC, EPEC, EIEC, ETEC, STEC)   Enteric virus as sole pathogen= 31 (RV, NV, Adenovirus, Astrovirus, Sapovirus) | **Microbiome composition in DEC group compared to viral and healthy samples:**   - At the phylum level, higher proportion of Proteobacteria and lower proportion of Firmicutes compared to viral and healthy groups, and higher proportion of Bacteroidetes compared to the healthy group. - At the family level, higher abundance of *Enterobacteriaceae* compared to viral and healthy groups - At the genus level, higher abundance of *Escherichia-Shigella* compared to viral and healthy groups, and also higher abundance of *Pseudocitrobacter* and *Bacteroides* compared to healthy group. - Indicative species for DEC group: *Escherichia albertii, Citrobacter werkmanii, Yersinia enterocolitica,* subsp*. paleartica*, and *Haemophilus sputorum* - Community structure analysis using redundancy analysis (RDA): different DEC pathotypes form independent clusters   **Microbiome composition in viral group compared to DEC and healthy groups:**   - Indicative species for viral group: *Holdemania filiformis, butyriciproducens, Christensenella minuta*, uncultured *Verrucomicrobiaceae*, [*Clostridium*] *lavalense* , *Eisenbergiella tayi*, uncultured *Subdoligranulum*, Blautia hansenii, *Hafnia alvei*, *Bacteroides dorei* - Community structure analysis using RDA: different enteric viruses do not form independent clusters. |
| Giggliucci et al. Front. Cell. Infect. Microbiol. 2018. | Italy | Fecal microbiome composition of samples from children 0-4 years of age collected during a STEC outbreak: Diarrheal samples (N=3), 2 weeks post diarrhea-recovery samples (N=3), and healthy controls (N=4)  Additionally, microbiome composition of stool samples from children 10-18 years of age with Crohn disease (N=4) was analyzed. | - Diarrheal samples collected during STEC outbreak were previously analyzed (diagnostic method not specified) and STEC O26:H11 was detected.  - Samples from patients with Crohn disease were analyzed by real-time PCR for the presence of virulence genes associated with STEC (stx1 and stx2) and EPEC (escV) | **Microbiome composition in STEC-samples compared to healthy-samples**   - Lower abundance of the members of *Bifidobacteriales* and Clostridiales orders, and higher abundance of *Lactobacillales* in comparison to controls. - *Enterococcus faecalis* and *Enterococcus avium* species, and the *Streptococcus* genus, belonging to the order *Lactobacillales*; and members of *Peptostreptococcaceae* and *Clostridiaceae* families from *Clostridiaceae* order predominated in the STEC-positive samples - *Lachnospiraceae* and *Ruminococcaceae* spp. (*Ruminococcus gnavus, Faecalibacterium prausnitzii*) from *Clostridiaceae* order were highly represented in healthy and post-diarrhea samples   **Microbiome composition in Crohn disease samples:**   - Intestinal dysbiosis characterized by lower complexity and depletion of benefitial *Bifidobacterium* species. Also, a high representation of *E. coli* species was found in 1 sample (positive for STEC-associated genes, both in real-time PCR and at the metagenomic analysis). |
| Chen et al. Scientific reports 2017. | Taiwan | Fecal microbiome composition analysis of samples from children hospitalized with severe or complicated viral acute gastroenteritis (N= 20, ages 0-91 months), and healthy controls (N=20, ages 0-54 months). | Diagnostic method is not specified.   - NV: 15 - RV: 5 | **Microbiome composition in diarrhea episodes according to clinical variables and compared to healthy controls:**   - Children with severe diarrhea had significantly decreased alpha-diversity score compared to healthy controls - Children with uncomplicated diarrhea had lower abundance of *Desulfovibrionaceae, Ruminococcaceae, Veillonellaceae* and increase in *Carnobacteriaceae* compared to healthy controls - Microbiome composition in controls and acute diarrhea had no differences at phylum level. At family level, controls had higher abundance of *Rikenellaceae* and *Porphyromonadaceae*, and *Alistipes* and *Parabacteroides* at genus level compared with any diarrhea   **Microbiome composition in acute diarrhea according to etiology:**   - Children with RV infection had a significantly higher alpha-diversity score compared to children with NV, and this latter was not different from controls.   **Microbiome composition according to clinical variables.**   - Children with severe diarrhea had significantly decreased alpha-diversity score compared to mild-moderate diarrhea - Children with complicated diarrhea had higher richness of *Campylobacteraceae*, *Neisseriaceae*, *Methylobacteriaceae, Sphingomonadaceae* and *Enterobacteriaceae* compared to healthy controls, and greater *Pasteurellaceae* richness compared to uncomplicated AGE. - Children with abdominal pain had higher abundance of *Prevotellaceae, Staphylococcaceae*, and *Coriobacteriaceae* at family level, and greater richness in *Prevotella*, TM7, *Staphylococcus* and *Atopobium* at genus level. - Children with extraintestinal manifestations of viral infection had lower abundance of *Micrococcaceae* and *Campylobacteraceae* - Children with convulsion had absence of *Haemophilus* and a substantial decrease of genus *Faecalibacterium.* |
| Mathew et al. Scientific Reports 2019. | Qatar | Fecal microbiome composition analysis of samples from children with diarrhea caused by viral or mixed virus-bacterial infection (N= 70, median age 14 months), and healthy controls (N= 9, median age 13.5 months) | FilmArray Gastrointestinal (GI) Panel kit®   - RV alone: 18 - RV + EAEC: 8 - RV + EPEC: 9 - RV + EPEC + EAEC: 5 - NV alone: 17 - NV + EAEC: 8 - NV + EPEC: 5 | **Microbiome composition in diarrhea groups compared to healthy controls:**   - Microbiome of healthy samples compared with RV and NoV groups differed in both diversity and composition - RV infection group had significantly scattered clustering compared to the controls, specially RV+EAEC and RV+EAEC+EPEC groups. NoV infection group showed microbe clustering compared to healthy samples with less significance than RV; higher significance in NV+ EAEC. - All RV infected groups had lower entropy scores compared to the control group, especially in those mixed with EAEC. NV infected groups had less divergent entropy scores. - Proportions of *Bacteroides* in the infected children was lower than controls, whereas *Bifidobacteriaceae* richness was more prominent in the bacterial-viral mixed infections and correlated with severity**.**   **Microbiome composition in RV group (alone and mixed with bacteria):**   - The most abundant genera in RV group were: *Bifidobacterium, Streptococcus, Escherichia, Bacteroides, Prevotella, Veillonella, Faecalibacterium, Clostridium, Collinsella* and unclassified genus of *Enterobacteriaceae, Veillonellaceae* and *Lachnospiraceae*. *Streptococcus, Escherichia, Prevotella* and *Veillonella* were the most prevalent and abundant - In RV+EAEC group: *Streptococcus* and Escherichia genera were also more prevalent and relatively abundant; while were less prevalent in RV+EPEC group - RV + EAEC + EPEC: predominant abundance of genera *Prevotella* and *Escherichia*   **Microbiome composition in NV group** **(alone and mixed with bacteria):**   - Most abundant genera in NV group were *Bifidobacterium, Bacteroides, Escherichia, Veillonella*, *Streptococcus, Faecalibacterium, Clostridium, Ruminococcus, Enterococcus* and unclassified genus of *Enterobacteriaceae, Veillonellaceae* and *Lachnospiraceae*. *Escherichia, Veillonella, Streptococcus, Faecalibacterium* and an unknown genus belonging to the *Lachnospiraceae* were highly prevalent across all NoV positive samples - NoV + EAEC and NV+EPEC groups: dominated by high levels of *Streptococcus, Escherichia* and *Clostridium* genera. Less abundance of *Enterococcus* and *Veillonellaceae* unclassified compared to total NV group.   **Microbiome composition and its relation with clinical variables:**   - The abundance of genus *Clostridium* resulted in increased frequency of diarrhea and vomiting in RV group - Children with fever had greater richness in *Prevotella* compared to other genera. - On the other hand, abundance of genus *Streptococcus* was observed to be associated with increase in diarrhea duration in NV group. - RV-alone group with an increased frequency of diarrhea exhibited greater abundance of Sulfur   River 1 , Lentisphaerae, Nitrospirae and Caldiserica , which were absent in NV-alone group- infected and healthy children   - Dominance of phyla Chlorobi was associated with moderate dehydration in NoV infected children - Richness of *Bifidobacteriaceae* increased with the severity of the viral-bacterial mixed infections. - RV+EAEC group: children with an onset of AGE symptoms for more than 2 days prior to hospitalization exhibited significant abundance and *Escherichia*, and lesser abundance of *Bacteroides* - NV + EAEC group: even lesser abundance of *Bacteroides*. Genera *Escherichia, Streptococcus, Rumella* and *Clostridium* were correlated with degree of dehydration - RV + EPEC group: children with high *Streptococcus* abundance had high frequency of vomits and diarrhea. - NV + EPEC groups: substantial decrease in genus *Bacteroides* and *Bifidobacterium* compared to RV+EPEC - Although mixed infection with EAEC resulted in significant microbiota differences compared to viral infection only or mixed infection with EPEC, the clinical condition of the children were worsened with both pathogenic *E.coli* co-infections |
| Gallardo P. et al. Front. Cell. Infect. Microbiol. 2020. | Chile | Fecal microbiome composition and metabolomics analysis from diarrheal samples of children positive for one DEC pathotype (N=8, ages 0-5 years) and healthy controls (N=8) | FilmArray® GI Panel   - Shigella/EIEC: 1 - STEC: 1 - EAEC: 3 - EPEC: 3 | **Microbiota composition in DEC group compared to healthy group:**   - Decrease in phyllum Firmicutes and increase in Bacteroidetes and Proteobacteria in DEC group compared to controls - Genera significantly associated with DEC group: *Gemella, Escherichia, Prevotella, Erwinia*, and *Buttiauxella* - Genera significantly associated with healthy group: *Faecalitalea*, *Lactococcus* and *Clostridium*.   **Metabolome in DEC group compared to healthy group:**   - Metabolic pathway predictions based on microbiota diversity showed that pathways involved in histidine degradation presented a higher representation of sequences associated with DEC group, while L-ornithine and L-histidine biosynthesis pathways were less represented in DEC group compared to healthy group - Metabolites analysis by LC-MS: higher levels of histamine and lower levels of ornithine in DEC samples. - Higher levels of histamine in DEC group: could be explained mainly by the presence of *Enterobacter hormaechei, Bifidobacterium stercoris, Shigella* spp., and *Citrobacter werkmanii/freundii* - Lower levels of ornithine in the DEC samples compared to healthy groups: could be explained mainly to the presence of *Streptococcus anginosus, Enterococcus faecalis* and *Escherichia* sp. |
| Xiong L et al. International Journal of Infectious Diseases 2021. | China | Fecal microbiome composition analysis of samples from children with diarrhea caused by RV (G9P8) (n=18, 11.8 ± 3.0 months) and NV (GII) (n= 24, 8.8 ± 6.4 months); and healthy controls (N= 25, ages 6.7 ± 4.3 mo.) | Diagnostic method is not specified.  - RV :18  - NV: 24 | **Clinical data:**   - Severity: 14 moderate cases and 45 severe cases. - Among RV: 90% were severe, NV: 57% were severe   **Microbiota composition in viral diarrhea compared to healthy controls:**   - Alpha-diversity: Chao1 index was higher in viral diarrhea compared to healthy controls. The Simpson indices of the RV and NV groups were lower than that of the control group, while there was no significant difference in Shannon index, although trends of reduction were observed in the viral groups, especially the NV group - Beta-diversity: Significant differences between the viral diarrhea and control groups - Infants with viral diarrhea showed lower relative abundance of Proteobacteria and higher relative abundances of Actinobacteria, Fusobacteria, Verrucomicrobia, and Cyanobacteria at the phylum level; and lower relative abundance of *Veillonella* and higher relative abundances of *Streptococcus* and *Enterococcus* at the genus level. - LEfSe analysis revealed the characteristic appearance of *Bacillus* among the intestinal microbiota of infants with viral diarrhea, both RV and NV, followed by *Streptococcus* and *Enterococcus*.   **RV v/s healthy group:**   - Higher abundance of Actinobacteria at the phylum level and higher abundances of *Bifidobacterium, Streptococcus, Enterococcus*, and *Lactobacillus* at the genus level   **NV v/s healthy group:**   - Higher abundances of Fusobacteria and Cyanobacteria at the phylum level and higher abundances of *Enterococcus* and *Streptococcus* at the genus level   **Microbiota composition in RV v/s NV**   - RV group had a lower alpha diversity than the NV group - No significant difference in beta diversity was observed between the RV and NV groups - At phylum level, the RV group exhibited higher abundances of Actinobacteria and Verrucomicrobia, while the NV group exhibited a higher abundance of Fusobacteria. At the genus level, the RV group showed higher abundances of *Veillonella* and *Bifidobacterium*, while the NV group showed higher abundances of *Enterococcus, Clostridium,* and *Fusobacterium*.   **Microbiota metabolism in viral diarrhea v/s controls:**   - Differences in aerobic, Gram-negative, Gram-positive and stress-tolerant bacteria between the viral and healthy groups. - NV group exhibited phenotypic differences in aerobic, anaerobic, mobile element–containing, Gram-negative, Gram-positive and stress-tolerant bacteria. - Compared with the control group, the viral group, the RV group, and the NV group all showed significant differences in potentially pathogenic bacteria - Viral group: upregulation of calcium signaling and photosynthesis in the viral infection groups, as well as marked upregulation of key metabolic pathways of biosynthesis, such as steroid biosynthesis, indole alkaloid biosynthesis, various types of N-glycan biosynthesis, and cellular functional pathways such as the apoptosis, cytochrome P450, and mRNA surveillance pathways. |

DEC: Diarrheagenic *Escherichia coli*

EAEC: Enteroaggregative *E. coli*

EIEC: Enteroinvasive *E. coli*

EPEC: Enteropathogenic *E. coli*

ETEC: Enterotoxigenic *E. coli*

LC-MS: Liquid chromatography–mass spectrometry

LEfSe: Linear discriminant effect-size analysis

NV: Norovirus

RDA: Redundancy analysis

RV: Rotavirus

STEC: Shiga toxic-producer *E. coli*
