# Supplementary material for: Bacterial Gut Microbiota and Infections During Early Childhood
Source: Front Microbiol. 2022 Jan 5;12:793050. doi: 10.3389/fmicb.2021.793050 (PMC8767011; doi:10.3389/fmicb.2021.793050)
Supplement: Supplementary file 2 [file Table_2.docx]

| **Supplementary Table 2: Studies assessing the bacterial gut microbiome composition during acute diarrhea episodes in children caused by any agent.** | | | | |
| --- | --- | --- | --- | --- |
| **Reference, country** | **Country** | **Study design, included patients** | **Etiology of diarrhea: diagnostic methods and detection rates** | **Results** |
| **Studies of microbiome and acute diarrhea in children caused by any agent.** | | | | |
| Pop et al. Genome Biology 2014. | West and East Africa,  and Southeast Asia | Comparison of fecal microbiota composition in children <5 years of age with moderate to severe diarrhea (N= 508), with microbiome of healthy controls (N= 484). | Etiology of diarrhea was not evaluated | **Microbiome composition in children with diarrhea:**   - Lower proportional abundance of obligate anaerobic microbes compared to controls - *Escherichia/ Shigella* and *Streptococcus* spp.: high proportion across all age groups, but their relative abundance decreases over time. *Prevotella* maintains an age-related increase of abundance (negative correlation of *Escherichia/Shigella* and *Prevotella* abundance) - Lower diversity compared to controls in every age group - Facultatively anaerobic or microaerophilic bacteria are associated with diarrhea: *Escherichia/Shigella, Granulicatella* spp., and *Streptococcus mitis/pneumonia* groups. Species *Escherichia/Shigella, Campylobacter jejuni*, and *Streptococcus pasteurianus*. Genera *Lactobacillus, Neisseria, Citrobacter, Erwinia,* and *Haemophilus*   **Microbiome functional prediction**   - Putative signatures of obligate anaerobic lineages associated with healthy status; and oxygen dependent pathways are enriched in diarrhea cases**.** |
| Kieser et al. Environmental Microbiology 2018. | Bangladesh | Comparison of fecal microbiota composition of children hospitalized with acute watery diarrhea (age 11.0 ± 4.1 months, N=71), and healthy controls (N=20). | Determined by standard methods:  - Only RV: 26  - RV + EAEC: 16  - Only EAEC: 6  - Only ETEC: 1  - Other bacteria: 7 (*Aeromonas*; *Aeromonas*  + RV; *Aeromonas* + *Vibrio*; *Vibrio*; *Vibrio* + ETEC;  *Campylobacter* + RV; *Shigella* + ETEC)  - Negative: 15 | **Microbiome composition in diarrhea cases compared to controls:**   - There was no consistent correlation between etiology diagnosis with standard methods and 16S rRNA gene sequencing - Lower α and within group β diversity than controls. - Higher abundance of *Streptococcus* compared to controls. At species level: *S. bovis* (including *S. equinus* and *S. gallolyticus*) and *S. Salivarius* - Decreased abundance of *Prevotella, Bifidobacterium, Bacteroides* and *Megamonas* compared with controls. *Escherichia* abundance was increased in all diarrhea groups except RV patients   **Metagenomic analysis:**   - **Controls:** higher mannan degradation capacity, enriched for methylerythritol phosphate pathway and the biosynthesis of thiamin (functions attributed to *Bacteroides*) - **Diarrhea cases:** enriched for a number of biosynthetic and degradation pathways without a common denominator including lactose and galactose degrading genes contributed by streptococci and anaerobic and to lesser extent aerobic respiration genes contributed by *E. Coli.* |
| Becker-Dreps et al. Am J Trop Med Hyg. 2015. | Nicaragua / USA | Comparison of fecal microbiota composition of children with acute diarrhea (N=25, mean age of 21.9 months) from a larger population-based study of infectious diarrhea etiologies, with a total of 41 episodes of acute diarrhea.  Diarrhea-free controls: stool collected from these children without diarrhea for at least 2 months. | Standard methods  **Diarrhea stools**: 61%: at least 1 enteropathogen detected (viral 32%, bacteria 15%, parasite 2%, and mixed 12%)  -Detected Virus: NV, Sapovirus, RV  -Detected Bacteria: ETEC, EHEC, *Shigella flexneri*, *Campylobacter* spp.  -Parasites: *Giardia lamblia*, *Cryptosporidium* spp., and *Entamoeba*  *histolytica/dispar*  **Recovery** **stools**: 44% with a pathogen detected. | **Microbiome composition in diarrhea cases compared to controls:**   - Phylogenetic diversity and species richness did not differ significantly between diarrhea and recovery stools - In those children with no bacteria detected during diarrhea episode, 59% presented an increase in diversity during recovery, while none children with any bacteria detected presents an increase in diversity. - Phylum Fusobacteria and Bacteroidetes were overrepresented in the diarrhea samples. *Cetobacterium* and *Fusobacterium* in the phylum Fusobacteria were overrepresented in the diarrhea group. Also, typical markers of a healthy gut microbiota *Achromobacter* and *Lactobacillus* were overrepresented in diarrhea group. |
| The et al. Gut Microbes 2017. | Vietnam / UK | Comparison of fecal microbiota composition of children <5 years of age with acute diarrhea with a confirmed infectious etiology prior  to antimicrobial therapy (N=145) and healthy control subjects (N=54) | Standard methods  Detection rate:   - NV: 13% - RV: 14% - *Campylobacter*: 10% - *Salmonella*: 10% - *Shigella*: 34% - *Campylobacter* + NV/RV: 5% - *Salmonella* + NV/RV: 3% - *Shigella* + NV/RV: 8% | **Microbiome composition in diarrhea cases compared to controls:**   - Fecal microbiota in diarrhea samples were categorized in 4 microbial configurations: 1.- *Bifidobacterium*-dominant, 2.- *Bacteroides*-dominant, 3.- *Streptococcus*-dominant, and 4.- *Escherichia*-dominant - Group 2 and controls had a higher Shannon diversity index compared to groups 1, 3 and 4 - Groups 1 and 3 (median 15-16 months) were associated with younger age compared to groups 2 and 4 (median 22-23 months) - Group 3 contained a significantly greater proportion of fecal samples with a bacterial pathogen detected - *Fusobacterium mortiferum* was the most significant species enriched in diarrheal samples compared to controls, while in lower significance also *Sutterella, Megamonas*, and *Enterococcus* species were enriched in diarrheal samples. - 19 OTUs were significantly depleted in diarrheal samples, most of them from the orders *Clostridiales* and *Erysipelotrichales*, including *Subdoligranulum, Roseburia, Eubacterium, Coprococcus, Catenibacterium,* and *Ruminococcaceae*. The most depleted OTU in all diarrheal groups compared to controls was *Blautia hansenii*, a known SCFA producer. - 19 OTUs were significantly enriched in bacterial infections in all diarrheal groups, including 8 taxa commonly associated with oral microbiota - 4 OTUs were enriched in viral diarrheas including *Bifidobacterium* species*: B. breve* and *B. Pseudocatenulatum.* - *B. Pseudocatenulatum, L. Reuteri* and *S. Gallolyticus* were depleted in dysenteric diarrhea. |
| **Studies of microbiome and acute diarrhea in children and adults, caused by any agent.** | | | | |
| Castaño-Rodríguez et al. Infect. Immun. 2018. | Australia | Fecal microbiota composition analysis in adults and children with acute gastroenteritis in a hospital-setting (N=475, ages: 1 month – 98 years) | Standard diagnostic methods   - Confirmed bacterial cases: 45   Confirmed viral cases: 27 | **Microbiome composition in diarrhea cases compared to controls:**   - Microbiome of diarrhea samples clustered into 3 groups: dominated by Bacteroidetes (*Bacteroides*), Proteobacteria (*Escherichia/Shigella*), or Firmicutes (*Faecalibacterium*). - In cases with no causative agent identified as etiology of diarrhea using standard methods, enriched bacterial taxa were detected, suggesting a utility of microbiome analysis to improve diagnosis. Proteobacteria dominant enterotype was no associated with detection of any specific agent as etiology of diarrhea - Microbial composition differed significantly at the OTU level between confirmed bacterial and viral cases. Specifically, significant differences in microbial compositions were observed between *C. difficile* and NV cases and *C. difficile* and RV cases but not between NV and RV.   **Microbiome functional prediction:**   - Subset of samples with a *Escherichia/Shigella*-dominated enterotype was more pro-inflammatory than the others according to metabolome prediction: pathways including bacterial invasion of epithelial, cells, drug metabolism - cytochrome P450, lipopolysaccharide biosynthesis proteins, RIG-I like receptor signaling pathway and glycan biosynthesis and metabolism. |
| Samb-Ba et al. PLoS ONE 2014. | Dakar, Senegal | Comparison of fecal microbiota composition of adults and children with diarrhea (N=347; 0-5 years old: 43.8%) and healthy controls (N=185) | Bacterial analysis was oriented to microbiome composition, and data about bacteria as etiology of diarrhea are not depicted.  Viruses and parasites analysis: macroscopic and microscopic  analyses  - RV: 10 (6.2%)  -Adenovirus: 4 (2.7%)  -RV+Adenovirus: 7 (4.3%)  -Parasites: 70 | **Microbiome composition in diarrhea cases compared to controls:**   - The number of bacterial species per fecal sample was significantly higher among patients without diarrhea than in those with diarrhea. - In patients with diarrhea, typically commensal bacteria were depleted compared to controls: *E. coli, Enterococcus* spp. (*E. faecium* and *E. casseliflavus*) and anaerobes, such as *Bacteroides* spp. (*B. uniformis* and *B. vulgatus*) and *Clostridium* spp. (*C. bifermentans, C. orbiscindens, C. perfringens, and C. symbosium*). - Bacterial species increased in diarrhea compared to controls: *Bacillus* spp. (*B. licheniformis, B. mojavensis,* and *B. pumilus* - For children from 0 to 5 year-old, 2 species of the genera *Clostridium* were significantly more frequent among those without diarrhea, including 1 species *C. glycolycum*, for which the data were not significant when the entire population was analyzed. |
| Braun et al. Scientific reports 2017. | Israel | Comparison of fecal microbiota composition of adults and children hospitalized with suspected infectious diarrhea (n=196) and in healthy, non-hospitalized adults (n = 881) | Stool culture  - Negative: 177  - *Campylobacter*: 14  - *Campylobacter* + *Salmonella*: 1  - *Salmonella*: 1  - *Shigella*: 3  Viruses and parasites were not analyzed. | **Microbiota composition in patients with diarrhea according to age**   - A significant increase in α-diversity was observed in the hospitalized patients during the first four years of life - Children <4 years old hospitalized with infectious diarrhea had higher abundance of taxa from Actinobacteria phyla and a lower abundance of the Bacteroidetes phyla compared to adults. At genus level, children had higher abundance of *Veillonella* and *Lactobacillus* (from Firmicutes phyla) and *Acinetobacter* (from Proteobacteria phyla).   **Microbiota composition in adults with diarrhea compared to healthy controls**.   - Significant increase in taxa from Proteobacteria phylum in comparison to healthy subjects, which is not explained by positive stool culture results. |
| Mizutani T et al. Scientific reports 2021. | Ghana | Comparison of fecal microbiota composition of adults and children with acute diarrhea and healthy adults.  -Total Cases: n = 80  -Children 0-10 years: n= n=18, median age: 3 years  - Adolescent 11–19 years: n= 13, median age: 15 years  - Adults: n= 49, median age 33 years. | Viral detection: RT-PCR  - NV: 39  - RV: 18  - No virus detected: 23 | **Gut microbiota profiles in diarrhea and controls:**   - Firmicutes, Proteobacteria, and Bacteroidetes were the predominant phyla in healthy adults and adolescents; and in adult and adolescent diarrheal patients. - Proteobacteria was the most dominant phylum in children with diarrhea - *Faecalibacterium*, *Subdoligranulum*, and *Escherichia-Shigella* were the dominant genera in healthy adults. *Escherichia-Shigella* were the most abundant genera among all ages in patients with diarrhea   **Gut microbiome composition in diarrhea and controls:**   - Relative abundances of the genera *Staphylococcus, Veillonella, Alloprevotella, Escherichia-Shigella,* and *Sutterella* were significantly elevated in adult patients with diarrhea. In contrast, *Faecalibacterium, Stenotrophomonas*, and *Subdoligranulum*, which are dominant genera in healthy Ghanaian adults, were significantly decreased in adult patients with diarrhea.   **Analysis for trends of causative pathogen in diarrheal patients.**   - *Escherichia-Shigella* was the most abundant genus in diarrheal stool samples, although *Escherichia-Shigella* is also among the top 3 genera in healthy Ghanaian adults. *Staphylococcus* and Salmonella were detected more frequently in patients with diarrhea than in healthy subjects; *Vibrio* and *Campylobacter* were detected at a high ratio in all age groups. *Aeromonas* was mainly detected in the stool samples of children - Diarrheal stools with NV or RV were found to have more bacteria at the genus level containing specific diarrheal causative bacteria than those of healthy subjects, suggesting the possibility of co‐infection   **Gut microbiome composition in viral v/s non-viral diarrhea:**   - **NV v/s non-viral:** At family level, increased proportions of *Erysipelotrichaceae* and *Staphylococcaceae*. At the genus level, increased *Holdemanella*, *Staphylococcus, Howardella, Corynebacterium* 1, and *Massilia* - **RV v/s non-viral**: no differences at family level. At genus level: *Acinetobacter* was increased, whereas *Dialister* and *Ruminococcaceae* NK4A214 group were decreased   **Functional and metabolic pathways in viral v/s non-viral diarrhea:**   - **RV v/s non-viral:** 10 pathways at KEGG level 2 were significantly different. The carbohydrate metabolism pathway to be different among the ten pathways (p = 0.002) 🡪 ascribed to alterations in fructose and mannose metabolism, pentose and glucuronate interconversions, galactose metabolism, and pyruvate metabolism - **NV v/s non-viral:** no differentially abundant KEGG pathway. |
| Singh et al. Microbiome (2015). Singh et al. Annals of Epidemiology 2016. | USA | Fecal microbiota composition of patients (adults and children) cursing diarrhea caused by *Campylobacter , Salmonella , Shigella*, and *STEC* (N=200), healthy family members (N=75) and patients post-infection (N=13) | Diagnostic method is not specified.  Etiology distribution:   - *Campylobacter* = 71 - *Salmonella* = 66 - *Shigella* = 34 - STEC = 28   Unknown= 1 | **Microbiome composition in diarrhea cases compared to healthy controls:**   - Lower species richness, evenness, and diversity in infected patients - Composition differences: mostly explained by differences in the abundance of phyla Proteobacteria, Bacteroidetes , and Firmicutes - Diarrhea cases had higher abundance of genus *Escherichia* (Proteobacteria), while healthy family members had a microbiota dominated by Bacteroides and Firmicutes   **Microbiome composition among diarrhea causes by different agents:**   - Clustering was not observed among patients infected with different pathogens, though significant differences were observed in the OTU composition and abundance across patients. - Proteobacteria dominated and comprised >20 % of the intestinal community in most diarrhea cases, and *Gammaproteobacteria* represented more than 90 % of the Proteobacteria detected - Hierarchical clustering indicates that microbiome composition does not varies significantly among patients infected with different pathogens   **Microbiome composition in post-diarrhea samples:**  Patients’ post-infection samples (up to 14 weeks post-recover) had a significant increase in Bacteroidetes and Firmicutes compared to infected samples, and clustered together with uninfected communities. |

EAEC: Enteroaggregative *E. coli*

ETEC: Enterotoxigenic *E. coli*

KEGG: Kyoto Encyclopedia of Genes and Genomes

NV: Norovirus

STEC: Shiga toxic-producer *E. coli*

RV: Rotavirus
